# Off‐season beach handball participation lowers injury incidence among handball players—A cross‐sectional survey on 641 athletes

**DOI:** 10.1002/ksa.12677

**Published:** 2025-04-18

**Authors:** Hannes Degenhardt, Maximilian Hinz, Benjamin D. Kleim, Marco‐Christopher Rupp, Alexander‐Stephan Henze, Sebastian Siebenlist, Romed P. Vieider, Yannick J. Ehmann

**Affiliations:** ^1^ Department of Sports Orthopaedics Technical University of Munich Munich Germany; ^2^ Department of Orthopedics Trauma Surgery and Sports Medicine, Muenchen Klinik Bogenhausen Munich Germany; ^3^ Department of Sports and Rehabilitation Medicine University Hospital Ulm Ulm Germany

**Keywords:** athletic injuries, beach handball, indoor handball, injury prevention, return to sport

## Abstract

**Purpose:**

To assess the effect of beach handball training during the indoor handball off‐season on the incidence of indoor handball injuries over three seasons. It was hypothesised that off‐season beach handball training would be associated with a lower injury incidence in indoor handball season.

**Methods:**

An online survey of indoor handball athletes was distributed between 04‐06/2023. Athletes' demographics, activity level, beach handball training during indoor handball off‐season, indoor handball injuries in the previous three years, injury mechanisms and time to return to sport were collected.

**Results:**

A total of 641 athletes (22 years [interquartile range [IQR]: 19–26]; 62.1% female) from 36 different countries were included. 50.1% (*n* = 321) played beach handball in the off‐season. The majority of athletes (92.7%, *n* = 594) played at a competitive (70.0%, *n* = 449) or a semi‐professional (22.6%, *n* = 145) level. During the three‐year period, 374 athletes (58.4%) reported a total of 501 injuries during indoor handball (incidence rate: 260.5 per 1000 athletes and year). The lower (66.1%) and upper extremity (26.3%) were the most frequently injured body parts. Overall, 87.0% (*n* = 436) of all athletes returned to indoor handball after injury. If participated in beach handball, a lower incidence of indoor handball injuries (odds ratio 0.60; [95% CI 0.42–0.87]; *p* = 0.006) and a lower injury rate in beach‐and‐indoor handball athletes was observed (225.3 vs. 295.8 injuries per 1000 athletes and year, *p* < 0.001).

**Conclusion:**

Participation in beach handball during indoor handball off‐season was associated with a significantly lower injury incidence during indoor handball. Beach handball may serve as a preventative training method to reduce injuries during breaks between indoor handball seasons.

**Level of Evidence:**

Level III, retrospective comparative study.

**Abbreviations**
ACL: anterior cruciate ligament
BMI: body mass index
CIs: confidence intervals
IQR: interquartile range
MCL: medical collateral ligament
ORs: odds ratios
RTS: return to sport
SD: standard deviation
SLAP: superior labrum anterior to posterior

## INTRODUCTION

Indoor handball is a physically demanding sport with a high risk of injury. Most injuries occur during contact situations with opposing players, particularly to the ankle, knee, shoulder, head and face [5, 16, 28]. In addition, overuse injuries, such as tendinopathies and stress fractures, have been reported due to the nature of the sport with repetitive stop‐and‐go movement patterns, contact situations and pivoting movements. Reducing the risk of these injuries requires comprehensive prevention, effective warm‐up routines and a precise execution of techniques [19, 21, 27].

Beach handball is similar to indoor handball, but is characterised by a sandy terrain, a smaller playing field, a smaller team size and unique rules that emphasise a non‐contact playing style. Beach handball is mostly played during the indoor handball off‐season and has gained popularity at beach sports events worldwide and has consequently been featured as an Olympic demonstration sport. Its injury patterns and mechanisms, risk factors and return to sport (RTS) dynamics have not yet been comprehensively investigated to date. Furthermore, the effects of beach handball training during the indoor handball off‐season on injury incidence are not well understood [2, 11]. There are indications that sand as a surface could have a preventive effect on injuries, as has been shown in volleyball and soccer, for example [17, 24, 25].

The present study aims to bridge these gaps in understanding the injury environment in beach handball and its relevance to injury prevention in indoor handball. Therefore, the study aimed to quantify the three‐year incidence of indoor handball‐related injuries and compare the results between indoor handball athletes who participated in beach handball during the indoor handball off‐season and those who did not. It was hypothesised that athletes that participate in beach handball during the indoor handball off‐season would have a lower incidence of indoor handball injuries and require surgery less frequently than athletes who do not play beach handball during the indoor handball off‐season.

## METHODS

### Survey composition and data collection

Approval for this cross‐sectional survey study was obtained by the institutional review board of the Technical University of Munich (reference: 654/20S). An interdisciplinary team consisting of sports medicine surgeons, sports medicine specialists and beach handball athletes and coaches developed a 47‐questions survey (see Supporting Information: Resource 1). Following a pilot phase, the final version of the questionnaire was administered via an online survey platform (SurveyMonkey Europe UC, Dublin, Ireland) between April and June 2023. The survey was conducted in English, and distribution occurred in two ways: First, the most frequently used social media platforms with handball and beach handball groups were targeted. Second, the questionnaire was distributed by email via handball team‐networks to reach indoor handball athletes. All data was collected anonymously. Incomplete surveys, those from athletes that only play beach handball and survey completed by underage athletes without parental consent were excluded from further analysis.

The survey comprised three sections. In the first section demographic data (e.g., gender, country of residence, age, height and weight) was collected. The second section focused on handball training experience and exposure (years of training, level of competition, practice and playing hours per week and competition frequency) in indoor and beach handball. In the third section, participants were asked to report any injuries they had sustained during the preceding three years of engaging in indoor handball. In the context of this study, an injury was defined as any musculoskeletal issue arising from training or competition in indoor handball. To exclude minor injuries, a significant injury was defined as an injury necessitating an absence from handball‐related training or competition for a minimum of seven days [12, 16]. All reported injuries were self‐disclosed and verification by a medical professional was not necessary for this study. For each reported injury, athletes were prompted to specify the anatomical location (head, neck, shoulder, upper arm, elbow, forearm, wrist, hand, finger, thorax and trunk, spine, upper back, lower back, hip, thigh, knee, calf, ankle and foot, toes) and the mechanism involved. Furthermore, athletes were asked to provide information on treatment and on the duration of time taken to resume full indoor handball training and competition following the injury.

### Statistical analysis

Statistical analysis was performed using the SPSS 26.0 software package (IBM‐SPSS, New York, USA). The level of significance was set at *p* < 0.05. Normal distribution was tested and graphically confirmed using the Kolmogorov–Smirnov test. After confirmation of normal distribution, continuous data were represented by means ± standard deviation (SD). Categorical variables were reported as frequencies and percentages. Non‐normally distributed continuous variables were presented as median and interquartile ranges (IQR). Normally distributed continuous variables between groups were compared using an unpaired two‐sample *t*‐test. Non‐normally distributed continuous variables and categorical variables between groups were compared using the Mann–Whitney *U* test. Associations between categorial variables were measured with Pearson's *χ*²‐test. Bonferroni correction and posthoc test were used for multiple pairwise comparisons. A multivariate logistic regression model was used to identify independent risk factors for sustaining injuries in indoor handball over the three‐year period prior to completion of the survey. The presence of an injury over the three‐year time period was defined as the dependent variable. Level of significance, adjusted odds ratios (ORs) and 95% confidence intervals (CIs) were calculated.

## RESULTS

### Study population

**Figure 1:**
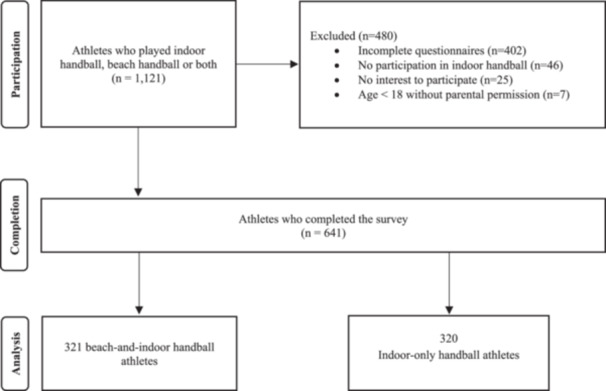
Flowchart of athletes' participation and distribution of the surveyed groups.

Out of a total of 1121 athletes, survey responses from 641 athletes (62.1% female) from 36 countries were eligible for final analysis (Figure 1). The demographic data are presented in Table 1, which shows no significant differences between injured and not‐injured athletes. However, significant differences are evident between male and female athletes in terms of age, weight, height and body mass index (BMI). Table 2 illustrates significant differences in the demographic data between beach‐and‐indoor handball athletes and indoor‐only handball athletes, particularly in relation to gender, weight and height. A more detailed comparison between female beach‐and‐indoor handball athletes and indoor‐only handball athletes reveals significant differences in height, as well as in BMI, when comparing male beach‐and‐indoor handball athletes with indoor‐only handball athletes (see Supporting Information: Resource 2 and 3).

**Table 1:** Demographics of injured versus not injured and male versus female handball athletes.

	All athletes (*n* = 641)	Injured athletes (*n* = 374)	Uninjured athletes (*n* = 267)	*p* value	Male athletes (*n* = 243)	Female athletes (*n* = 398)	*p* value
Sex							
Male	243 (37.9%)	131 (35.0%)	112 (41.9%)	> 0.05	—	—	—
Female	398 (62.1%)	243 (65.0%)	155 (58.1%)	> 0.05	—	—	—
Age, y (IQR)	22 (19–26)	22 (19–25)	22 (18–28)	> 0.05	23 (19–28)	22 (19–25)	0.007*
Height, cm (IQR)	175 (170–184)	175 (170–184)	176 (170–185)	> 0.05	186 (180–190)	172 (168–176)	< 0.001*
Weight, kg (IQR)	72 (64–82)	72 (65–82)	72.0 (63–84)	> 0.05	85 (76–92)	67 (61.8–73)	< 0.001*
BMI (IQR)	23.3 (21.5–25.2)	22.8 (20.3–24.8)	23.0 (21.7–25.0)	> 0.05	24.5 (22.6–26.3)	22.7 (21.2–24.2)	< 0.001*

*Note*: Non‐normally distributed continuous are shown as median and interquartile ranges (IQR), categorical variables are shown as number of patients and percentages per group. Bolded *p*‐values and asterisks indicates significant difference between groups (*p* < 0.05).

Abbreviation: BMI, body mass index.

**Table 2:** Demographics of beach‐and‐indoor handball athletes versus indoor‐only handball athletes.

	Beach‐and‐indoor handball athletes (*n* = 321)	Indoor‐only handball athletes (*n* = 320)	*p* value
Sex			
Male	161 (50.2%)	82 (25.6%)	< 0.001*
Female	160 (49.8%)	238 (74.4%)	< 0.001*
Age, y (IQR)	22 (19–27)	23 (19–26)	> 0.05
Height, cm (IQR)	179 (172–186)	174 (168–180)	< 0.001*
Weight, kg (IQR)	75 (65–85)	69 (62.25–80)	< 0.001*
BMI (IQR)	23.4 (21.7–25.2)	23.1 (21.3–25.2)	> 0.05

*Note*: Non‐normally distributed continuous are shown as median and interquartile ranges (IQR), categorical variables are shown as number of patients and percentages per group. Bolded *p*‐values and asterisks indicates significant difference between groups (*p* < 0.05).

Abbreviation: BMI, body mass index.

The athletes' data on years playing indoor handball, playing hours per week and off‐season training are presented in Table 3. No significant differences were identified between injured and not injured athletes. Beach‐and‐indoor handball athletes spent substantially more time per week in indoor handball (*p* = 0.002), competing at a higher level of play, with a notably increased presence in nationwide leagues (*p* < 0.001), while engaging in fewer endurance training sessions in off‐season (*p* < 0.001) compared to indoor‐only handball athletes. Male athletes performed significantly less endurance training than female athletes (*p* = 0.009; Supporting Information: Resource 4). Furthermore, male beach‐and‐indoor handball athletes played significantly more hours per week than male indoor‐only handball athletes (*p* = 0.001) and female beach‐and‐indoor handball athletes engage significant less in endurance training than female indoor‐only handball athletes in the off‐season (*p* = 0.01), see Supporting Information: Resource 5).

**Table 3:** Exposure, level of play and off‐season training of all athletes.

	All athletes (*n* = 641)	Injured athletes (*n* = 374)	Uninjured athletes (*n* = 267)	*p* Value	Beach‐and‐indoor handball athletes (*n* = 321)	Indoor‐only handball athletes (*n* = 320)	*p* value	
Exposure								
Years (IQR)	15 (10–18)	15 (11–19)	14 (10–18)	> 0.05	14 (10–18)	15 (11–18)	> 0.05	
Hours per week (IQR)	7 (5–10)	7 (5–9.3)	7 (5–10)	> 0.05	7 (5–10)	6 (5–8)	0.002*	
Level of play, *n* (%)								
Professional	35 (5.5)	22 (5.9)	13 (4.9)	> 0.05	22 (6.9)	13 (4.1)	> 0.05	
Semi‐professional	145 (22.6)	90 (24.1)	55 (20.6)	> 0.05	77 (24.0)	68 (21.3)	> 0.05	
Competitive	449 (70.0)	255 (68.2)	194 (72.7)	> 0.05	220 (68.5)	229 (71.6)	> 0.05	
Amateur	12 (1.9)	7 (1.9)	5 (1.9)	> 0.05	2 (0.6)	10 (3.1)	> 0.05	
Highest level of competition in last 3 years, *n* (%)								
International	71 (11.1)	39 (10.4)	32 (12.0)	> 0.05	41 (12.8)	30 (9.4)	> 0.05	
Nationwide	204 (31.8)	122 (32.6)	82 (30.7)	> 0.05	123 (38.3)	81 (25.3)	< 0.001*	
Regional	282 (44.0)	170 (45.5)	112 (41.9)	> 0.05	123 (38.3)	159 (49.7)	< 0.001*	
Local	84 (13.1)	43 (11.5)	41 (15.4)	> 0.05	34 (10.6)	50 (15.6)	> 0.05.	
Off‐season training (between two indoor handball seasons), *n* (%)								
Strength	424 (66.1)	261 (69.8)	163 (61.0)	> 0.05	208 (64.8)	216 (67.5)	> 0.05	
Flexibility	284 (44.3)	177 (47.3)	107 (40.1)	> 0.05	135 (42.1)	149 (46.6)	> 0.05	
Endurance	482 (75.2)	291 (77.8)	191 (71.5)	> 0.05	223 (69.5)	259 (80.9)	< 0.001*	
Neuromuscular	131 (20.4)	80 (21.4)	51 (19.1)	> 0.05	68 (21.2)	63 (19.7)	> 0.05	
No Sports	59 (9.2)	31 (8.3)	28 (10.5)	> 0.05	34 (10.6)	25 (7.8)	> 0.05	

*Note*: Non‐normally distributed continuous are shown as median and interquartile ranges (IQR), categorical variables are shown as number of patients and percentages per group. Bolded *p*‐values and asterisks indicates significant difference between groups (*p* < 0.05).

### Injury incidence

A total of 501 injuries (female athletes: 327, male athletes: 174) were reported with an incidence rate of 260.5 injuries per 1,000 athletes and year. Overall, 374 athletes (58.4%) reported injuries within the examined time period which led to an absence of training for more than one week. Of these, 261 athletes (40.7%) reported one injury, 99 athletes (15.4%) reported two injuries, and 14 athletes (2.2%) reported three injuries. A total of 267 athletes (41.7%) remained injury‐free with no significant differences between male and female athletes (male: 112, female: 155, *p* > 0.05). No significant gender‐specific differences were observed in the number of injured athletes (female: 243, 61.1%, male: 131, 53.9% *p* > 0.05), the number of multiple injuries (*p* > 0.05), or the location of injuries (*p* > 0.05).

When comparing injury incidence of beach‐and‐indoor handball athletes and indoor‐only handball athletes, injury incidence was 225.3 versus 295.8 injuries per 1000 athletes per year (*p* < 0.001) with significantly more injured indoor‐only handball athletes than beach‐and‐indoor handball athletes (208; 65.0% vs. 166; 51.7%; *p* < 0.001). There was a significantly lower total number of injuries in beach‐and‐indoor handball athletes (*n* = 217) compared to indoor‐only handball athletes (*n* = 284) overall with a lower injury rate per player (0.68 vs. 0.89, *p* < 0.001). When considering the number of injuries per athlete, beach‐and‐indoor handball athletes reported less injuries in each category: one injury (118; 36.8% vs. 143; 44.7%; *p* > 0.05), two injuries (45; 14.0% vs. 54; 16.9%; *p* > 0.05) and three injuries (3; 0.9% vs. 11; 3.4%; *p* = 0.01). There were significantly more beach‐and‐indoor handball athletes who stayed injury free than indoor‐only handball athletes (155; 48.3% vs. 112; 35.0%; *p* < 0.001), respectively.

In total, a higher percentage of male indoor‐only handball athletes were injured than male beach‐and‐indoor handball athletes (50, 61.0%, vs. 81, 50,3%; *p* > 0.05) with a higher percentage of injuries (0.83 vs. 0.66 injuries per player; *p* > 0.05) with no statistical significance. There was no significant difference in the body region affected by injury comparing male beach‐and‐indoor handball athletes and male indoor‐only handball athletes (*p* > 0.05). Overall, significantly more female indoor‐only handball athletes were injured than beach‐and‐indoor handball athletes (158, 66.4% vs. 85, 53.1%; *p* = 0.005) with significantly higher total numbers of injuries (216 vs. 111) and a higher injury rate per player (0.91 vs. 0.69; *p* = .003). There were no statistical differences in the location of injuries between female beach‐and‐indoor handball athletes and indoor‐only handball athletes (*p* > 0.05).

The most frequently injured body regions of injuries were the lower extremity (66.1%) and the upper extremity (26.3%). Considering distribution between beach‐and‐indoor handball and indoor‐only handball athletes, the lower extremity was more frequently injured in indoor‐only handball athletes than in beach‐and‐indoor handball athletes (137; 63.1% vs. 194; 68.3%; *p* > 0.05). Tables 4 and 5 provide a comprehensive breakdown of injury locations for beach‐and‐indoor handball and indoor‐only handball athletes, as well as injuries sustained by male and female athletes. Of all injuries, female athletes experienced a higher incidence of knee/calf/lower leg injuries than male athletes (118, 36.1% vs. 45, 25.9%; *p* > 0.05). Female indoor‐only handball athletes reported more injuries to the knee/calf/lower leg (82, 38.0% vs. 36, 32.4%; *p* > 0.05) and shoulder (30, 13.9% vs. 11, 9.9%; *p* > 0.05) and fewer injuries to the hand/wrist (16, 7.4% vs. 14, 12.6%; *p* > 0.05) compared to female beach‐and‐indoor handball athletes (Supporting Information: Resource 6). Male indoor‐only handball athletes indicated more injuries to the shoulder (13, 19.1% vs. 11, 10.4%; *p* > 0.05) and fewer injuries to the hand/wrist (4, 5.9% vs. 15, 14.2%; *p* > 0.05) than male beach‐and‐indoor handball (Supporting Information: Resource 7). In particular, beach‐and‐indoor handball athletes suffered fewer superior labrum anterior to posterior (SLAP) lesions than indoor‐only handball athletes (female: 0, 0.0% vs. 9, 4.2%; *p* > 0.05; male: 1, 0.9% vs. 4, 5.9%; *p* > 0.05). Regarding injuries to the anterior cruciate ligament (ACL), female beach‐and‐indoor handball reported fewer ACL sprains (1, 0.9% vs. 7, 3.2%; *p* > 0.05) and ACL tears (12, 10.8%, vs. 28, 13.0%; *p* > 0.05) than indoor‐only handball athletes (Supporting Information: Resource 8).

**Table 4:** Distribution of body region affected by Injury in beach‐and‐indoor handball athletes versu indoor‐only handball athletes.

	Total injuries (*n* = 501)	Beach‐and‐indoor handball athletes (*n* = 217)	Indoor‐only handball athletes (*n* = 284)	*p* value
Location of Injury, *n* (%)				
Head/neck	27 (5.4)	14 (6.0)	13 (4.6)	> 0.05
Chest wall/torso/abdomen	4 (.8)	3 (1.4%)	1 (.4)	> 0.05
Spine (below Neck)	9 (1.8)	4 (1.8)	5 (1.8)	> 0.05
Shoulder	65 (13.2)	22 (10.1)	43 (15.1)	> 0.05
Elbow/arm	17 (3.4)	9 (4.2)	8 (2.8)	> 0.05
Hand/wrist	49 (9.8)	29 (13.4)	20 (7.0)	> 0.05
Hip/pelvis/thigh	28 (5.6)	14 (6.5)	14 (4.9)	> 0.05
Knee/calf/lower leg	163 (32.5)	64 (29.5)	99 (34.9)	> 0.05
Ankle or foot	139 (27.7)	58 (26.7)	81 (28.5)	> 0.05

*Note*: Categorical variables are shown as number of patients and percentages per group.

**Table 5:** Distribution of body region affected by injury in in male versus female athletes.

	Total injuries (*n* = 501)	Injuries of male athletes (*n* = 174)	Injuries of female athletes (*n* = 327)	*p* value
Location of Injury, *n* (%)				
Head/neck	27 (5.4)	6 (3.5)	21 (6.4)	> 0.05
Chest wall/torso/abdomen	4 (.8)	3 (1.7)	1 (0.3)	> 0.05
Spine (below neck)	9 (1.8)	1 (0.6)	8 (2.5)	> 0.05
Shoulder	65 (13.2)	24 (13.8)	41 (12.5)	> 0.05
Elbow/arm	17 (3.4)	7 (4.0)	10 (3.1)	> 0.05
Hand/wrist	49 (9.8)	19 (10.9)	30 (9.2)	> 0.05
Hip/pelvis/thigh	28 (5.6)	16 (9.2)	12 (3.7)	> 0.05
Knee/calf/lower leg	163 (32.5)	45 (25.9)	118 (36.1)	> 0.05
Ankle or foot	139 (27.7)	53 (30.5)	86 (26.3)	> 0.05

*Note*: Categorical variables are shown as number of patients and percentages per group.

### Risk factors

An independent significant risk factor for sustaining an injury was a longer indoor handball career (OR 1.05; [95% CI 1.01–1.10]; *p* = 0.02), with a 5% increase in the odds for injury for each additional year of play. The participation in beach handball during the off‐season led to a significant reduction in the odds for injury in indoor handball (OR 0.60; [95% CI 0.42–0.87]; *p* = 0.006). There was 20.2% reduction in the likelihood of injury in indoor handball among athletes that participated in beach handball in the previous off‐season, with a risk ratio of 0.798. For detailed data see Supporting Information: Resource 9.

### Injury mechanism

Regarding injury mechanisms, 38.9% were caused by contact situations (*n* = 195), particularly with other athletes (157, 31.3%), while 22.2% of all injuries occurred after a jump (*n* = 111). Beach‐and‐indoor handball athletes suffered significantly less injuries caused by contact situations with other athletes than indoor‐only handball athletes (59; 27.2% vs. 104; 36.6%; *p* = 0.03; Supporting Information: Resource 10). No statistical difference was found between male and female athletes (58, 33.3% vs. 105, 32.1%, *p* > 0.05) and female beach‐and‐indoor handball athletes and indoor‐only handball athletes in terms of contact situations with other athletes (31; 27.9% vs. 74; 34.3%; *p* > 0.05). In the male subgroup however, beach‐and‐indoor handball athletes suffered significantly less injuries caused by contact situations with other athletes than indoor‐only handball athletes (28; 26.4% vs. 30; 44.1%; *p* = 0.016).

### Injury timing and position played

Of all injuries, 53.5% occurred during competition with 21.4% in first two months, 36.3% in middle, and 23.8% in last two months of the regular season. The majority of injuries occurred in offence (56.9%; 26.1% in defence). Back court players were the most affected (53.1%). Notably, there were significantly less injuries among athletes in the pivot position which played beach handball during off‐season (18; 8.3% vs. 54; 19.0%; *p* < 0.001; see Supporting Information: Resource 11). Specifically, male pivot players that played beach handball suffered significantly fewer injuries (4, 3,7% vs. 15, 22.1%, *p* < 0.001). No statistical difference was found between female beach‐and‐indoor handball athletes and indoor‐only handball athletes regarding the position played. There was no difference in the injury incidence of beach‐and‐indoor athletes that completed a transition training (see Supporting Information: Resource 12). Transition training is a parallel structured beach handball training conducted in the lasts months of the indoor handball season, as well as beach handball training at the start of the upcoming indoor handball season. No statistical difference was found between male and female beach‐and‐indoor handball athletes and indoor‐only handball athletes in terms of injury timing, competition or training related injuries and offence or defence (*p* > 0.05).

### Treatment of injuries

In terms of treatment, 67.9% of the injuries were treated conservatively with rest (63.7%), physiotherapy (59.7%), immobilisation or brace‐based stabilisation (41.1%) and regular painkillers (31.9%), while 32.1% received surgery. Beach‐and‐indoor handball athletes reported a significantly lower intake of regular painkillers (55; 25.3% vs. 105; 37.0%; *p *< 0.001) and required less surgery (54; 24.9% vs. 105; 37.0%; *p* < 0.001), see Supporting Information: Resource 13. Female athletes used significantly more regular painkillers (118, 36.1% vs. 42, 24.1%; *p* = 0.007) and required surgery more frequently than male athletes (117, 35.8% vs. 40, 23.0%; *p* = 0.004). Female beach‐and‐indoor handball athletes played with a stabilising brace significantly more often than indoor‐only handball athletes (17, 15.3% vs. 64, 29.6%, *p* = 0.005) and required less surgery (32, 28.8% vs. 87, 40.3%; *p* = 0.003). Male beach‐and‐indoor handball athletes used significantly fewer regular painkillers than indoor‐only handball athletes (18, 17.0% vs. 24, 35.3%; *p* = 0.005).

### Return to sports

After injury, 87.0% (*n* = 436) of athletes returned to indoor handball, while 9.6% (*n* = 48) of the injuries were recent and a complete recovery had not yet been possible. The remaining 3.4% of all athletes (*n* = 17) suffered 12 knee injuries (1x unhappy triad, 4x ACL tear, 1x medical collateral ligament (MCL) tear, 3x meniscal tears and 4x tendinitis), two shoulder injuries (1x unstable shoulder and 1x impingement syndrome), one ankle injury (syndesmosis injury) and one hand injury (subluxated wrist). The reasons for not returning were pain (*n* = 8), the fear of getting injured again (*n* = 3), persisting instability (*n* = 2), inability to gain previous form (*n* = 2) and not specified reasons (*n* = 2). Within 4 weeks, 29.5% of the athletes returned to full volume and intensity of indoor handball and in total 51.4% did so within 2 months. Significantly more injured beach‐and‐indoor handball athletes returned within the first four weeks (70; 37.0% vs. 54; 21.9%; *p* < 0.001; see Supporting Information: Resource 14). No statistically significant differences were found between female and male athletes, female beach‐and‐indoor handball athletes and indoor‐only handball athletes and male beach‐and‐indoor handball athletes and indoor‐only handball athletes in terms of return to full indoor handball and time to return to full indoor handball (*p* > 0.05).

## DISCUSSION

### Key findings

The most important finding of this study was that athletes who play beach handball during the indoor handball off‐season reported significantly lower injury rates in indoor handball. Furthermore, beach‐and‐indoor handball athletes suffered significantly less injuries caused by contact situations than indoor‐only handball athletes and reported significantly fewer required surgeries with earlier return to full indoor handball.

### Injury incidence, location and type of Injury

Indoor‐only handball athletes experienced significantly more frequent and overall injuries, particularly in the lower extremities, compared to those also participating in beach handball, with no significant gender differences observed. In accordance to the existing literature in terms on the higher risk for severe knee injuries in female athletes, our results indicate a trend towards more knee injuries in female athletes in general, and particularly in female indoor‐only handball athletes, who sustained more anterior cruciate ligament tears and sprains than female beach‐and‐indoor handball athletes [18, 20, 29]. These results suggest that the distinctive training environment and physical demands of beach handball may contribute to improved lower extremity joint stability, which could potentially reduce the risk of injuries in these areas. The variation in terrain, such as sand courts, may enhance proprioception, acceleration, jumping, sprint speed, change of direction speed and lower limb adaptability and thus may be advantageous with regard to injury prevention [8, 22]. Moreover, the dynamic nature of sand courts may stimulate greater lower extremity muscle engagement, thereby promoting joint stability and potentially reducing injury rates [26]. Binnie et al. [6] observed a significant increase in VO_2_ max following an 8‐week training programme on sand in comparison to grass in netball and field hockey players. Furthermore, it has been shown that single‐leg jumps onto sand show a positive effect on knee abduction moment and anterior‐posterior tibial translation. Sand training reduces knee abduction moment during drop jumps and anterior‐posterior tibial translation during level jumps, lowering stress on the knee's medial structures and potentially reducing ACL injury risk [25].

Sport‐specific training on sand is associated with reduced musculoskeletal stress, increased motor unit recruitment and a more favourable cardiovascular training effect [7, 10, 13, 23]. Studies that have compared the injury patterns and epidemiology of beach volleyball and indoor volleyball have identified that the different playing surfaces, environmental conditions, and variations in game dynamics between the two disciplines are associated with different injury patterns and less injuries in beach volleyball [1, 3, 15, 26].

In addition to the benefits for the lower extremities, the present study also revealed a significantly lower incidence of shoulder injuries among beach‐and‐indoor handball. This reduction in injuries was observed among those who continued to engage in sport‐specific training through beach handball during the indoor handball off‐season. The diversity of training environments and playing conditions in beach handball including throwing and shoulder exercises appears to foster enhanced shoulder joint stability, muscle coordination and strength. These findings show the potential of beach handball training to protect shoulder health and reduce the incidence of shoulder‐related injuries among indoor handball athletes, as has been shown in other sports played on the beach [4].

### Injury mechanisms

Beach handball athletes, especially males, experienced fewer contact‐related injuries than indoor‐only athletes. This could be an indication of an adapted playing style due to the non‐contact rules as well as improved positional play, improved anticipation, the permanent numerical advantage in offence as well as improved timing and faster transition play in beach handball [14]. Due to the absence of studies on the effects of beach handball training on indoor handball, sport‐specific injury prevention measures should still be emphasised. Both indoor and beach handball may benefit from injury prevention programmes tailored to the sport's demands and common injury patterns [19, 28].

### Injury timing and playing position

With regard to the timing of injury, no relevant difference can be identified between the indoor‐only handball athletes and beach‐and‐indoor handball athletes. When comparing beach handball players who have completed structured transition training, where beach handball was practiced concurrently with the indoor handball season and vice versa, it was found that athletes with transition training are more likely to be injured in the pre‐season, but less likely to be injured in the last two months of the regular season. This temporal difference in injury occurrence may be attributed to the unique physical demands of beach handball during the summer months, followed by the transition to indoor handball. Coaches and medical staff should consider implementing targeted preseason conditioning programmes for beach handball athletes to reduce the early‐season injury risk, potentially addressing the transition challenges more effectively. Especially as transition training has been shown to improve jumping performance without negatively impacting indoor performance [3, 9].

Furthermore, the study showed an advantageous effect of beach handball training on pivot position athletes, who experienced significantly fewer injuries, highlights the potential benefits of beach handball in enhancing the physical attributes and skills required for this specific position in indoor handball. Future research could delve deeper into the specific training methodologies and biomechanical factors contributing to this advantage, providing valuable insights for athlete development and position‐specific training.

### Treatment and return to full indoor handball

Beach‐and‐indoor handball athletes reported fewer injuries, lower painkiller use, quicker recovery, and better rehabilitation, suggesting that beach handball's training and conditioning may reduce serious injuries and enhance recovery. This finding underline the importance of optimising rehabilitation protocols and physical conditioning strategies in both indoor and beach handball.

### Clinical relevance

The clinical relevance of this study lies in presenting the potential preventative effects of participating in beach handball between the indoor handball seasons. These findings suggest that including beach handball as an injury prevention strategy for indoor handball athletes could reduce the risk of injury in indoor handball [2]. With indoor handball being a popular and physically demanding sport, the findings of reduced injury rates in individuals incorporating beach handball training offer valuable insights for sports medicine practitioners, coaches, and athletes. By integrating beach handball into the training schedule, coaches and staff may reduce the occurrence and severity of injuries.

## LIMITATIONS

This study has several limitations: First, this study uses retrospective data, where all injuries, diagnoses, severity level, and return to activity/sport/competition time frames were self‐reported without confirmation by a medical professional. This means that the accuracy of the reported injuries and diagnoses may be limited. Second, the analysis of return to sport is limited by including recent injuries that are still in the healing process during data collection, making it difficult to distinguish them from injuries from which athletes may never fully recover. Third, the questionnaire was only available in English imposing a potential language barrier for non‐native speakers. Fourth, we set a maximum limit of three reportable severe injuries in this study. Although this was done to minimise the time required to complete the survey and maintain high participation rates, it may have resulted in under‐reporting of injuries for some athletes who suffered more than three injuries. Nevertheless, only 14 athletes (2.2%) reported three injuries. Fifth, the dropout rate (*n* = 480, 42.8%) during the answering of the questionnaire may impose a selection bias. However, making the questionnaire longer the authors were able to gain deeper insights in the reported injuries. Sixth, it is possible that already injured or injury‐prone indoor handball athletes do not play beach handball during off‐season, so that beach handball could be collectively considered healthier.

## CONCLUSION

Participation in beach handball during indoor handball off‐season was associated with a significantly lower injury incidence during indoor handball. In addition, injuries sustained in indoor handball in athletes who participated in beach handball in the off‐season required surgery less frequently and had a shorter time to return to sport. Beach handball may serve as a preventative training method to reduce injuries during breaks between indoor handball seasons.

## AUTHOR CONTRIBUTIONS

All authors contributed to the study conception and design. Hannes Degenhardt made substantial contributions to data acquisition, interpretation, drafting, and revising the manuscript. Maximilian Hinz, Benjamin D. Kleim, and Marco‐Christopher Rupp were involved in data interpretation, drafting the manuscript, and adding intellectual content to the final paper. Alexander‐Stephan Henze contributed significantly to data acquisition, data interpretation, drafting the manuscript, and adding intellectual content. Sebastian Siebenlist made substantial contributions to the conception, acquisition, and drafting of the paper, as well as adding important intellectual content to the final manuscript. Romed P. Vieider played a vital role in data acquisition, analysis, and drafting the manuscript and was significantly involved in statistical analysis. Yannick J. Ehmann was crucial for data acquisition, study design, and conception and was critical in drafting the manuscript and interpreting the data. All authors read and approved the final manuscript.

## CONFLICT OF INTEREST STATEMENT

The authors, Hannes Degenhardt, Maximilian Hinz, Benjamin D. Kleim, Marco‐Christopher Rupp, Alexander‐Stephan Henze, Romed P. Vieider, and Yannick J. Ehmann declare no conflicts of interest. Sebastian Siebenlist has received consultant fee payments from Arthrex GmbH, KLS Martin Group and medi GmbH & Co. KG unrelated to this study.

## ETHICS STATEMENT

The Ethics Committee of the Technical University of Munich approved the conduct of this study (Project Number: 654/20 S). Consent was obtained from all participants before their participation.

## Supporting information

ESM 1.

ESM 2 clean.

ESM 3 clean.

ESM 4 clean.

ESM 5 clean.

RevisedESM 6.

RevisedESM 7.

ESM 8.

ESM 9.

RevisedESM 10.

ESM 11.

ESM 12.

ESM 13.

ESM 14.

## Data Availability

The data sets used and/or analysed during the current study are available from the corresponding author on reasonable request.
