# Supplementary material for: Off‐season beach handball participation lowers injury incidence among handball players—A cross‐sectional survey on 641 athletes
Source: Knee Surg Sports Traumatol Arthrosc. 2025 Apr 18;33(6):2307–16. doi: 10.1002/ksa.12677 (PMC12104784; doi:10.1002/ksa.12677)
Supplement: Supplementary file 1 — ESM 1. [file KSA-33-2307-s013.docx]

Online Resource 1

# Participation

1. Would you like to participate?

- Yes
- No

1. If you are <18 years old, are your parents informed and do they agree to your participation in this survey? (requirement)

- I’m over 18 years old
- Yes, my parent(s) or guardian agree to my anonymous participation
- No

# Demographic data

1. What is your biological gender?

- Male
- Female

1. How old are you?

1

100


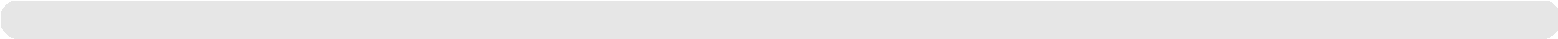

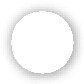

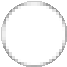


1. In which country do you live?

- Austria
- Angola
- Argentina
- Brazil
- Belarus
- Croatia
- Czech Republic
- Denmark
- Egypt
- France
- Germany
- Hungary
- Iceland
- Japan
- Korea
- Macedonia
- Latvia
- Netherlands
- Norway
- Qatar
- Portugal
- Poland
- Russia
- Slovenia
- Sweden
- Switzerland
- Serbia
- Spain
- Ukraine
- USA

Others

1. How tall are you in cm? (Note: 5“0 = 153 cm; 5“2 = 158 cm; 5“4 = 163 cm; 5“6 = 168 cm 5“8 = 173 cm; 5“10 = 178 cm; 6”0 = 183 cm; 6”2 = 188 cm; 6”4 = 193 cm; 6“6 = 198 cm; 6“8 = 203 cm; 6“10 = 208 cm; 7“0 = 213 cm)

1

250


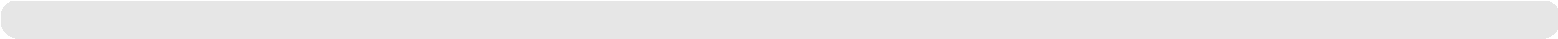

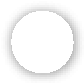

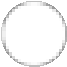


1. How much do you weigh in kg? (Note: One pound = 0.45 Kilograms)

1

180


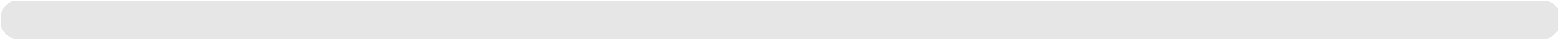

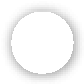

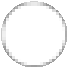


# Sporting activity

1. Do you play Indoor/Team Handball?

- Yes
- No

[If questions 8 is answered “no”, end of survey]

# Indoor/Team handball

1. For how many years have you been playing indoor/team handball regularly?

1

100


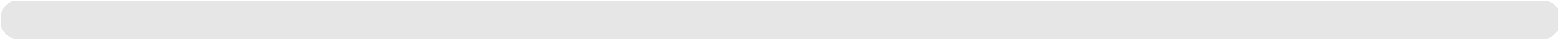

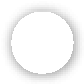

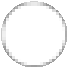


1. How many hours do you play/train indoor/team handball per week?

1

100


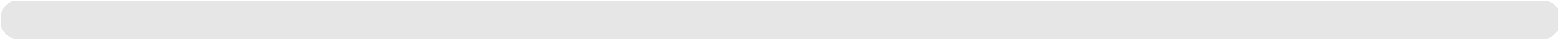

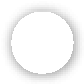

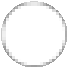


1. At what level do you play?

- I play casually (not in a team)
- I play at a competitive level (in a team)
- I play at a semi-professional level (in a team, receiving money/sponsorship/scholarship)
- I am a professional indoor/team handball player (indoor/team handball is my main occupation)

1. What was the highest level you competed at in the last 5 years?

- Local competitions
- Regional competitions
- Nationwide competitions
- International competitions

# Offseason

1. How long in weeks was the overall season break from indoor handball (regardless of what you did during the season break)?

1

100


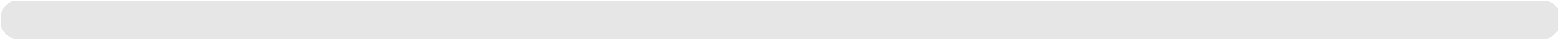

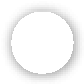

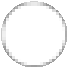


1. Do you regularly play beach handball? If injured, did you play in the off-season before the injury?

- Yes
- No

1. What else do you regularly do in off-season?

- Strength training
- Flexibility training
- Endurance training
- Neuromuscular/proprioceptive training
- No sports, regeneration
- Other sports (please specify)

# Beachhandball (If q14 is answered with “yes”)

1. For how many years have you been playing beach handball regularly?

1

100


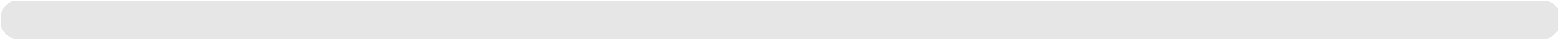

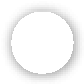

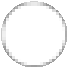


1. How many months a year do you play/train beach handball?

1

100


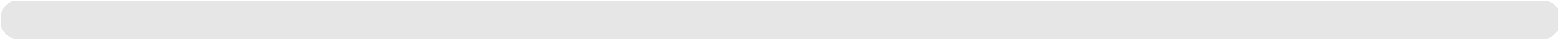

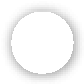

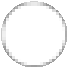


1. How many hours do you play/train beach handball per week when competing?

1

100


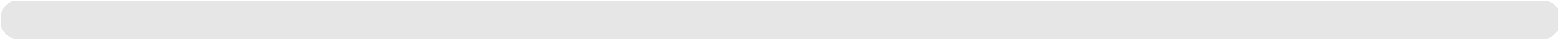

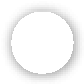

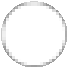


1. What position do you usually play in beach handball? (please tick all that apply)

- Goalkeeper
- Defense
- Specialist/Shooter
- Left Wing
- Right wing
- Pivot
- Backfield

1. At what level do you play?

- I play casually (not in a team)
- I play at a competitive level (in a team)
- I play at a semi-professional level (in a team, receiving money/sponsorship/scholarship)
- I am a professional beach handball player (beach handball is my main occupation)

1. What was the highest level you competed at in the last 5 years?

- Local competitions
- Regional competitions
- Nationwide competitions
- International competitions

1. Was a transition training performed from indoor handball to beach handball and vice versa? (Transitional training is the gradual increase of the beach handball training sessions during the end of the indoor handball season (for example in the last 6 weeks of the season) and vice versa.

- Yes
- No

# Transition training

1. *(If q22 yes)* How many weeks was a transition training performed from indoor season to beach handball season?

1

100


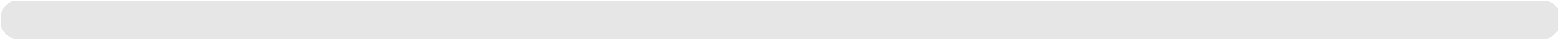

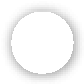

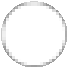


1. How many weeks was a transition training performed from beach season to indoor handball season?

1

100


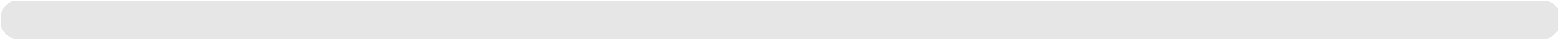

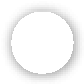

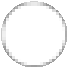


# Injuries

1. Have you sustained an acute injury (with a sudden onset of pain/symptoms, associated with one specific identifiable traumatic event (e.g. twisting joint, collision, etc.) **while / from playing indoor/team handball** in the last **three years**?

- Yes
- No

1. *(If q25: yes) Did this injury render you unable to play and practice* indoor/team handball *for a minimum of 7 days?*

- Yes
- No

# Acute Injuries

## Type

1. Please indicate the location of your injury (choose one)

- Head and neck
- Spine (below neck)
- Chest Wall / Torso / Abdomen
- Shoulder
- Elbow / arm
- Hand / wrist
- Hip / pelvis/ thigh
- Knee /calf / lower leg
- Ankle or foot

[Forward to one of questions 31 to 39 depending on the selected location]

1. What kind of head or neck injury did you have?

- *Head:* Broken nose
- *Head:* Broken bone of the face or skull (other than nose)
- *Head: Eye injury*
- *Brain:* Concussion
- *Brain:* Traumatic brain injury with permanent damage
- *Brain:* loss of consciousness (with symptoms possibly lasting for up to 2 weeks)
- *Neck:* Broken vertebrae (bone of the neck)
- *Neck:* Whiplash injury
- *Neck:* Disk injury
- *Neck:* Nerve injury (with loss of sensation (feeling) or muscle power)
- *Soft tissue:* Laceration (wound) of skin
- *Others:* Chipped/broken tooth
- Face injury, not specified
- Eye injury, not specified
- Skull injury, not specified
- Neck injury, not specified

1. What kind of spine injury (below the neck) did you have?

- Broken vertebra (bone)
- Sacral stress fracture
- Disk injury / prolapse
- Muscular back pain
- Contusion / Bruising
- Ligament sprains / whiplash
- Spinal nerve injury with a loss of sensation (feeling) or muscle power
- Laceration/ Skin wound
- Upper back injury, not specified
- Lower back injury, not specified
- Rib injury, not specified
- Spine injury, not specified

1. What kind of torso / chest wall / abdominal injury did you have?

- *Bone:* Rib fracture
- *Bone:* Rib contusion / bruise
- *Joint:* Sternoclavicular joint dislocation/instability (inner collar bone joint injury)
- *Muscle:* Pectoralis major/minor muscle injury
- *Lung:* Pneumothorax (air leaked outside of the lungs)
- *Abdominal:* Injury to a solid organ (that is, liver, spleen, pancreas, or kidneys)
- Abdominal: Injury to a hollow organ (that is, stomach, small intestine, colon, or bladder)
- Abdominal: Injury to the abdominal muscles
- Injury to reproductive organs
- Laceration / Skin wound
- Chest injury, not specified
- Abdominal injury, not specified

1. What kind of shoulder injury did you have?

- *Bone:* Broken humerus (fracture of upper arm at the joint)
- *Bone:* Broken scapula (shoulder blade)
- *Bone:* Broken clavicle (collarbone)
- *Joint:* Dislocated shoulder
- *Joint:* Subjectively „unstable“ shoulder / subluxation
- *Joint:* Separated shoulder (acromioclavicular joint injury/dislocation)
- *Joint:* Sternoclavicular joint dislocation/instability (inner collar bone joint injury)
- *Joint:* Bursitis / tendinitis of the rotator cuff
- *Muscle:* Pectoralis major/minor muscle injury
- *Tendon:* Rotator cuff tear
- *Tendon:* Impingement Syndrome
- *Tendon:* SLAP-tear (tear of the upper glenoid labrum - rim around the socket - where the long head of biceps tendon attaches)
- *Tendon:* Long head of the Biceps tendinitis
- *Functional:* Scapular dyskinesia / malposition / “SICK shoulder” (movement disorder or asymmetry of shoulder bone)
- *Nerve:* Suprascapular neuropathy / palsy (atrophy of infraspinatus muscle with decreased external rotation force
- *Soft tissue:* Muscle strain/tear
- *Soft tissue:* Laceration (wound) of skin
- *Soft* tissue: Contusion / bruise
- Shoulder injury, not specified

1. What kind of elbow or arm injury did you have?

- *Bone:* Broken ulna (forearm)
- *Bone:* Broken radius (forearm)
- *Bone:* Broken humerus (upper arm, excluding shoulder region)
- *Joint:* Elbow dislocation
- *Ligament:* Radial Collateral Ligament (RCL) injury
- *Ligament:* Thrower's elbow (sprain injury of the ulnar collateral ligament)
- *Tendon:* Epicondylitis / tennis or golfer‘s elbow
- *Tendon:* Biceps tendon rupture (at the elbow)
- *Tendon:* Biceps tendonitis (at the elbow)
- *Tendon:* Triceps tendon rupture
- *Tendon:* Triceps tendonitis
- *Nerve:* Ulnar nerve inflammation
- *Soft tissue:* Muscle strain/tear
- *Soft tissue:* Laceration / Skin wound
- *Soft tissue:* Contusion / bruise
- Elbow injury, not specified
- Upper arm injury, not specified
- Forearm injury, not specified

1. What kind of acute hand or wrist injury did you have?

- *Bone:* Broken ulna (forearm bone at the wrist)
- *Bone:* Broken radius (forearm bone at the wrist)
- *Bone:* Broken carpal bone at the wrist (small bones of the hand)
- *Bone:* Broken metacarpal bone (long bones in the palm of the hand)
- *Bone:* Broken finger
- *Joint:* Wrist sprain
- *Joint:* Finger dislocation
- *Joint:* Jammed finger
- *Base of the thumb:* Gamekeeper's thumb / skier's thumb / UCL tear (injury to the ulnar collateral ligament (UCL)
- *Tendon:* Mallet finger / hammer finger (inability to straighten the fingertip due to an extensor tendon injury)
- *Tendon:* Jersey finger / rugby finger (Inability to flex the fingertip due to a flexor tendon injury)
- *Nerve:* Palm nerve injury (De Quervain Tenosynovitis)
- *Soft tissue: Laceration / Skin wound*
- *Wrist / metacarpal injury, not specified*
- *Finger injury, not specified*

1. What kind of hip / pelvic / thigh injury did you have?

- *Bone:* Broken femur (thigh bone)
- *Bone:* Broken pelvic bone
- *Bone:* Tailbone injury
- *Joint:* Labral tear (cartilage rim around hip socket)
- *Joint:* Femoroacetabular impingement (deformity of the neck of the femur)
- *Muscle:* Pulled/ Torn/ Strained hamstring muscle
- *Muscle:* Avulsion / tear off from the bone of the quadriceps muscle at the hip
- *Soft tissue:* Runners hip (trochanteric bursitis at the outside of the hip)
- *Soft tissue:* Snapping hip syndrome / dancer's hip (inflammation and snapping sensation when bending and stretching the hip)
- *Soft tissue:* Piriformis syndrome (compression of the sciatic nerve by the piriformis muscle)
- Pelvis injury, not specified
- Hip injury, not specified
- Thigh injury, not specified

1. What kind of knee or lower leg injury did you have?

- *Bone:* Broken femur (thigh)
- *Bone:* Broken tibia (lower leg)
- *Bone:* Broken fibula (lower leg)
- *Ligament:* Anterior cruciate ligament (ACL) **tear**
- *Ligament:* Posterior cruciate ligament (PCL) **tear**
- *Ligament:* Medial/inner collateral ligament (MCL) **tear**
- *Ligament:* Lateral/outer collateral ligament (LCL) **tear**
- *Ligament:* Anterior cruciate ligament (ACL) **sprain**
- *Ligament:* Posterior cruciate ligament (PCL) **sprain**
- *Ligament:* Medial/inner collateral ligament (MCL) **sprain**
- *Ligament:* Lateral/outer collateral ligament (LCL) **sprain**
- *Ligament:* Runner’s knee / Iliotibial ligament syndrome (inflammation on the outside of the knee due to friction between the iliotibial ligament and the outside femur)
- *Joint:* Torn meniscus
- *Joint:* Cartilage injury
- *Joint:* Kneecap dislocation
- *Tendon:* Patellar tendinitis/ jumper’s knee
- *Tendon:* Pes anserinus syndrome (inflammation of the tendon attachments at the lower inner side of the knee)
- *Muscle:* Quadriceps tendon (thigh muscle) tear at the knee
- *Muscle:* Calf muscle injury
- Knee injury, not specified
- Lower leg injury, not specified

1. What kind of ankle / foot injury did you have?

- *Bone:* Broken tibia (lower leg bone at the ankle)
- *Bone:* Broken fibula (lower leg bone at the ankle)
- *Bone:* Broken bone of the foot
- *Bone:* Broken toe
- *Joint:* Cartilage injury
- *Ligaments:* Ankle **sprain** lateral / outside
- *Ligaments:* Ankle **sprain** medial / inside
- *Ligaments:* Outer/ Lateral ankle ligament **tear** or bony avulsion
- *Ligaments:* Inner/ Medial ankle ligament **tear** or bony avulsion
- *Tendon:* Achilles tendinitis (inflammation)
- *Tendon:* Achilles tendon rupture
- *Soft tissue:* Sand toe (hyperflexion/ downward-flexion of the toe, resulting in an injury of the dorsal/upper joint capsule)
- Ankle injury, not specified
- Foot injury, not specified
- Toe injury, not specified

## Risk factors

1. What mechanism caused your injury?

- *Jump:* Coming down on the floor after a jump
- *Jump:* Coming down on a team mate after a jump
- *Jump:* Coming down on an opponent after a jump
- *Contact:* Hit by a team mate coming down after a jump
- *Contact:* Hit by an opponent coming down after a jump
- *Contact:* Collision with a team mate
- *Contact:* Collision with an opponent (offense)
- *Contact:* Collision with an opponent (defense)
- *Contact:* Contact with the ball
- *Contact:* Contact with the floor
- *Contact:* Contact with the goal
- *Contact:* Contact with out-of-bounds apparatus
- *Playing the ball:* Whilst blocking
- *Playing the ball:* Whilst passing
- *Playing the ball:* Whilst throwing
- *Playing the ball:* Whilst tipping / bouncing
- *Others:* Unintentionally hit by ball
- *Others:* Falling
- *Others:* Rapid change of direction
- *Others:* Rotation around planted foot
- *Others:* Acute overwork
- Others: (please specify)

1. What position did you play when you got injured?

- Goalkeeper
- Back court center
- Back court right
- Back court left
- Left wing
- Right wing
- Pivot

1. Did this injury occur during training or competition?

- Training
- Competition

1. Did this injury occur during offense or defense?

- Offense
- Defense
- Dose not apply

1. When was the injury (in indoor handball)?

- During preseason
- In the first two months of regular season
- In the middle of the regular season
- In the mid break of the season
- In the last two months of the season
- During off-season

1. How was your injury treated? (please tick all that apply)

- Rest / Waited it out (missed training and competitions)
- Regular painkillers
- Anti-inflammatory medication
- Injections/ infiltrations (e.g. into torn muscle or into inflamed area)
- Physiotherapy
- Stabilization in a brace
- Immobilization in a Splint
- Immobilization in a Cast
- Surgery
- Other (please specify)

1. At the time when this injury was worst, did you have any difficulties in normal training and competition due to the injury?

- Full participation without health problems
- Full participation, but with symptoms from the injury
- Reduced participation due to the injury
- Could not participate due to the injury

1. Have you returned to playing indoor/team handball since your injury?

- Yes
- No

1. *(if q44: no)* What has kept you from going back?

- My injury was recent, I’m still expecting to return
- Pain
- Loss of range of motion (of a joint)
- Persisting instability
- Fear of getting injured again
- Loss of position on team
- Unable to regain previous form
- Doctor’s advice

1. How long did it take to return to full indoor/team handball training / competition?

- My injury did not make me stop indoor/team handball training / competition at any point
- Less than 1 week
- 1 – 4 weeks
- ~ 2 months
- ~ 3 months
- ~ 4 months
- ~ 5 months
- ~ 6 months
- ~ 7 months
- ~ 8 months
- ~ 9 months
- ~ 10 months
- ~ 11 months
- ~ 12 months
- > 1 year
- I did not return yet, but I think I will
- I did not return and I don’t think I will

# Further injury

1. Did you have another acute injury (with a sudden onset of pain/symptoms, associated with one specific identifiable traumatic event (e.g. wrong movement, collision, etc.)) whilst playing indoor/team handball in the last three years?

- Yes.
- No.

[Repeat questions 29 through 47 until the answer to question 47 is “no”.]
