# Supplementary material for: Off‐season beach handball participation lowers injury incidence among handball players—A cross‐sectional survey on 641 athletes
Source: Knee Surg Sports Traumatol Arthrosc. 2025 Apr 18;33(6):2307–16. doi: 10.1002/ksa.12677 (PMC12104784; doi:10.1002/ksa.12677)
Supplement: Supplementary file 2 — ESM 2 clean. [file KSA-33-2307-s001.docx]

Online Resource 2: Demographics of female beach-and-indoor handball athletes vs. indoor-only handball athletes

|  | Demographics of female beach-and-indoor handball athletes vs. indoor-only handball athletes | | | |
| --- | --- | --- | --- | --- |
|  | Female athletes (n=398) | Female Beach-and-indoor handball athletes (n=160) | Female Indoor-only handball athletes  (n=238) | *P*-value |
| Age, y (IQR) | 22 (19-25) | 22 (19-25) | 22 (19-25) | > .05 |
| Height, cm (IQR) | 172 (168-176) | 172 (169-177) | 171 (167.8-175) | **.02*** |
| Weight, kg (IQR) | 67 (61.8-73) | 68 (62-75) | 67 (61-72) | > .05 |
| BMI (IQR) | 22.7  (21.2-24.2) | 22.6 (21.0-24.2) | 22.7 (21.2-24.3) | > .05 |

Non-normally distributed continuous are shown as median and interquartile ranges (IQR), categorical variables are shown as number of patients and percentages per group. Bolded p-values and asterisks indicates significant difference between groups (p< .05).
