# Supplementary material for: Off‐season beach handball participation lowers injury incidence among handball players—A cross‐sectional survey on 641 athletes
Source: Knee Surg Sports Traumatol Arthrosc. 2025 Apr 18;33(6):2307–16. doi: 10.1002/ksa.12677 (PMC12104784; doi:10.1002/ksa.12677)
Supplement: Supplementary file 3 — ESM 3 clean. [file KSA-33-2307-s007.docx]

Online Resource 3: Demographics of male beach-and-indoor handball athletes vs. indoor-only handball athletes

|  | Demographics of male beach-and-indoor handball athletes vs. indoor-only handball athletes | | | |
| --- | --- | --- | --- | --- |
|  | Male athletes (n=243) | Male Beach-and-indoor handball athletes (n=161) | Male Indoor-only handball athletes  (n=82) | *P*-value |
| Age, y (IQR) | 23 (19-28) | 23 (18.5-29) | 24 (20.8-27) | > .05 |
| Height, cm (IQR) | 186 (180-190) | 186 (180-191) | 185.5 (180-190) | > .05 |
| Weight, kg (±SD) | 85 (76-92) | 84 (76-91.5) | 88 (75-93.5)) | > .05 |
| BMI (IQR) | 24.5 (22.6-26.3) | 24.3 (22.6-25.6) | 25.0 (23.0-27.1) | **.047*** |

Normally distributed continuous variables are shown as mean ± standard deviation, non-normally distributed continuous are shown as median and interquartile ranges (IQR), categorical variables are shown as number of patients and percentages per group. Bolded p-values and asterisks indicates significant difference between groups (p< .05).
