# Supplementary material for: Off‐season beach handball participation lowers injury incidence among handball players—A cross‐sectional survey on 641 athletes
Source: Knee Surg Sports Traumatol Arthrosc. 2025 Apr 18;33(6):2307–16. doi: 10.1002/ksa.12677 (PMC12104784; doi:10.1002/ksa.12677)
Supplement: Supplementary file 4 — ESM 4 clean. [file KSA-33-2307-s014.docx]

Online Resource 4: Exposure, Level of Play and Preventative Measures of male vs. female athletes

|  | All athletes (n=641) | Male athletes (n=243) | Female athletes (n=398) | P-value |
| --- | --- | --- | --- | --- |
| Exposure |  |  |  |  |
| Years (IQR) | 15 (10-18) | 14 (10-19) | 15 (11-18) | > .05 |
| Hours per Week (IQR) | 7 (5-10) | 8 (5-10) | 6 (5-9) | > .05 |
| Level of Play, n (%) | |  |  |  |
| Professional | 35 (5.5) | 17 (7.0) | 18 (4.5) | > .05 |
| Semi-professional | 145 (22.6) | 56 (23.1) | 89 (22.4) | > .05 |
| Competitive | 449 (70.0) | 168 (69.1) | 281 (70.6) | > .05 |
| Amateur | 12 (1.9) | 2 (0.8) | 10 (2.5) | > .05 |
| Highest Level of Competition in Last 3 Years, n (%) | | | |  |
| International | 71 (11.1) | 34 (14.0) | 37 (9.3) | > .05 |
| Nationwide | 204 (31.8) | 83 (34.2) | 121 (30.4) | > .05 |
| Regional | 282 (44.0) | 96 (39.5) | 186 (46.7) | > .05 |
| Local | 84 (13.1) | 30 (12.4) | 54 (13.6) | > .05 |
| Off-Season Training (Between Two Indoor Handball Seasons), n (%) | | | | |
| Strength | 424 (66.1) | 166 (68.3) | 258 (64.8) | > .05 |
| Flexibility | 284 (44.3) | 96 (39.5) | 188 (47.2) | > .05 |
| Endurance | 482 (75.2) | 169 (69.6) | 313 (78.6) | **.009*** |
| Neuromuscular | 131 (20.4) | 42 (17.3) | 89 (22.4) | > .05 |
| No Sports | 59 (9.2) | 24 (9.9) | 35 (8.8) | > .05 |

Non-normally distributed continuous are shown as median and interquartile ranges (IQR), categorical variables are shown as number of patients and percentages per group. Bolded p-values and asterisks indicates significant difference between groups (p< .05).
