# Supplementary material for: Off‐season beach handball participation lowers injury incidence among handball players—A cross‐sectional survey on 641 athletes
Source: Knee Surg Sports Traumatol Arthrosc. 2025 Apr 18;33(6):2307–16. doi: 10.1002/ksa.12677 (PMC12104784; doi:10.1002/ksa.12677)
Supplement: Supplementary file 5 — ESM 5 clean. [file KSA-33-2307-s011.docx]

Online Resource 5: Exposure, Level of Play and Preventative Measures of male beach-and-indoor handball athletes vs. indoor-only handball athletes and female beach-and-indoor handball athletes vs. indoor-only handball athletes

|  | All athletes (n=641) | Male beach-and-indoor handball athletes (n=161) | Male indoor-only handball athletes (n=82) | | P-value | | Female beach-and-indoor handball athletes (n=160) | | Female indoor-only handball athletes (n=238) | *P*-value |  |
| --- | --- | --- | --- | --- | --- | --- | --- | --- | --- | --- | --- |
| Exposure |  |  |  | |  | |  | |  |  |  |
| Years (IQR) | 15 (10-18) | 15 (10-19) | 14 (10-18.3) | | > .05 | | 14 (11-18) | | 15 (11-18.3) | > .05 |  |
| Hours per Week (IQR) | 7 (5-10) | 8 (6-10) | 6 (4-8) | | **.001*** | | 7 (5-10) | | 6 (5-9) | > .05 |  |
| Level of Play, n (%) | |  |  | |  | |  | |  |  |  |
| Professional | 35 (5.5) | 15 (9.3) | 2 (2.4) | | > .05 | | 7 (4.4) | | 11 (4.6) | > .05 |  |
| Semi-professional | 145 (22.6) | 40 (24.8) | 16 (19.5) | | > .05 | | 37 (23.1) | | 52 (21.9) | > .05 |  |
| Competitive | 449 (70.0) | 105 (65.2) | 63 (76.8) | | > .05 | | 115 (71.9) | | 166 (69.8) | > .05 |  |
| Amateur | 12 (1.9) | 1 (0.6) | 1 (1.2) | | > .05 | | 1 (0.6) | | 9 (3.4) | > .05 |  |
| Highest Level of Competition in Last 3 Years, n (%) | | | |  | |  | |  | |  |  |
| International | 71 (11.1) | 27 (16.8) | 7 (8.5) | | > .05 | | 14 (8.8) | | 23 (9.7) | > .05 |  |
| Nationwide | 204 (31.8) | 63 (39.1) | 20 (24.4) | | > .05 | | 60 (37.5) | | 61 (25.6) | > .05 |  |
| Regional | 282 (44.0) | 54 (33.5) | 42 (51.2) | | > .05 | | 69 (43.1) | | 117 (49.2) | > .05 |  |
| Local | 84 (13.1) | 17 (10.6) | 13 (15.9) | | > .05 | | 17 (10.6) | | 37 (15.6) | > .05 |  |
| Off-Season Training (Between Two Indoor Handball Seasons), n (%) | | | | | | | | | | | |
| Strength | 424 (66.1) | 107 (66.5) | 59 (72.0) | | > .05 | | 101 (63.1) | | 157 (66.0) | > .05 |  |
| Flexibility | 284 (44.3) | 60 (37.3) | 36 (43.9) | | > .05 | | 75 (46.9) | | 113 (47.5) | > .05 |  |
| Endurance | 482 (75.2) | 107 (66.5) | 62 (75.6) | | > .05 | | 116 (72.5) | | 197 (82.8) | **.01*** |  |
| Neuromuscular | 131 (20.4) | 29 (18.0) | 13 (15.9) | | > .05 | | 39 (24.4) | | 50 (21.0) | > .05 |  |
| No Sports | 59 (9.2) | 17 (10.6) | 7 (8.5) | | > .05 | | 17 (10.6) | | 18 (7.6) | > .05 |  |

Non-normally distributed continuous are shown as median and interquartile ranges (IQR), categorical variables are shown as number of patients and percentages per group. Bolded p-values and asterisks indicates significant difference between groups (p< .05).
