# Supplementary material for: Off‐season beach handball participation lowers injury incidence among handball players—A cross‐sectional survey on 641 athletes
Source: Knee Surg Sports Traumatol Arthrosc. 2025 Apr 18;33(6):2307–16. doi: 10.1002/ksa.12677 (PMC12104784; doi:10.1002/ksa.12677)
Supplement: Supplementary file 6 — RevisedESM 6. [file KSA-33-2307-s010.docx]

Online Resource 6: Distribution of Injury Location in female beach-and-indoor handball athletes vs. female Indoor-only handball athletes

|  | | | | |
| --- | --- | --- | --- | --- |
|  | All injuries of female athletes (n=327) | Injuries of female beach-and-indoor handball athletes (n=111) | Injuries of female Indoor-only handball athletes (n=216) | P-value |
| Location of Injury, n (%) |  |  |  |  |
| Head/Neck | 21 (6.4) | 10 (9.0) | 11 (5.1) | > .05 |
| Chest Wall/Torso/Abdomen | 1 (0.3) | 0 (0.0) | 1 (0.5) | > .05 |
| Spine (below Neck) | 8 (2.5) | 4 (3.6) | 4 (1.9) | > .05 |
| Shoulder | 41 (12.5) | 11 (9.9) | 30 (13.9) | > .05 |
| Elbow/Arm | 10 (3.1) | 4 (3.6) | 6 (2.8) | > .05 |
| Hand/Wrist | 30 (9.2) | 14 (12.6) | 16 (7.4) | > .05 |
| Hip/Pelvis/Thigh | 12 (3.7) | 5 (4.5) | 7 (3.3) | > .05 |
| Knee/Calf/Lower leg | 118 (36.1) | 36 (32.4) | 82 (38.0) | > .05 |
| Ankle or Foot | 86 (26.3) | 27 (24.4) | 59 (27.3) | > .05 |

*Categorical variables are shown as number of patients and percentages per group. Bolded p-values* *and asterisks indicates significant difference between groups (p< .05).*
