# Supplementary material for: Off‐season beach handball participation lowers injury incidence among handball players—A cross‐sectional survey on 641 athletes
Source: Knee Surg Sports Traumatol Arthrosc. 2025 Apr 18;33(6):2307–16. doi: 10.1002/ksa.12677 (PMC12104784; doi:10.1002/ksa.12677)
Supplement: Supplementary file 7 — RevisedESM 7. [file KSA-33-2307-s012.docx]

Online Resource 7: Distribution of Injury Location in male beach-and-indoor handball athletes vs. male Indoor-only handball athletes

|  | | | | |
| --- | --- | --- | --- | --- |
|  | All injuries of male athletes (n=174) | Injuries of male beach-and-indoor handball athletes (n=106) | Injuries of male Indoor-only handball athletes (n=68) | P-Value |
| Location of Injury, n (%) |  |  |  |  |
| Head/Neck | 6 (3.5) | 4 (3.8) | 2 (2.9) | > .05 |
| Chest Wall/Torso/Abdomen | 3 (1.7) | 0 (0.0) | 3 (4.4) | > .05 |
| Spine (below Neck) | 1 (0.6) | 0 (0.0) | 1 (1.5) | > .05 |
| Shoulder | 24 (13.8) | 11 (10.4) | 13 (19.1) | > .05 |
| Elbow/Arm | 7 (4.0) | 5 (4.7) | 2 (2.9) | > .05 |
| Hand/Wrist | 19 (10.9) | 15 (14.2) | 4 (5.9) | > .05 |
| Hip/Pelvis/Thigh | 16 (9.2) | 9 (8.5) | 7 (10.3) | > .05 |
| Knee/Calf/Lower leg | 45 (25.9) | 28 (26.4) | 17 (25.0) | > .05 |
| Ankle or Foot | 53 (30.5) | 31 (29.3) | 22 (32.4) | > .05 |

*Categorical variables are shown as number of patients and percentages per group. Bolded p-values* *and asterisks indicates significant difference between groups (p< .05).*
