# Supplementary material for: Off‐season beach handball participation lowers injury incidence among handball players—A cross‐sectional survey on 641 athletes
Source: Knee Surg Sports Traumatol Arthrosc. 2025 Apr 18;33(6):2307–16. doi: 10.1002/ksa.12677 (PMC12104784; doi:10.1002/ksa.12677)
Supplement: Supplementary file 8 — ESM 8. [file KSA-33-2307-s005.docx]

Online Resource 8: Diagnosis of injuries and distribution between beach-and-indoor handball athletes vs. indoor-only handball athletes

|  | | | | |  |
| --- | --- | --- | --- | --- | --- |
| Location, n (%) | All injuries (n=501) | Injuries of beach-and-indoor handball athletes (n=217) | Injuries of indoor-only handball athletes  (n=284) | p-value | |
| **Knee / Calf / Lower leg** | **163 (32.5)** | **64 (29.5)** | **99 (34.9)** | > .05 | |
| Ligament: Anterior cruciate ligament (ACL) tear | 52 (10.4) | 18 (8.3) | 34 (12.0) | > .05 | |
| Joint: Torn meniscus | 27 (5.4) | 10 (4.6) | 17 (6.0) | > .05 | |
| Knee injury, not specified | 16 (3.2) | 8 (3.7) | 8 (2.8) | > .05 | |
| Tendon: Patellar tendinitis/ jumper’s knee | 10 (2.0) | 4 (1.8) | 6 (2.1) | > .05 | |
| Ligament: Anterior cruciate ligament (ACL) sprain | 8 (1.6) | 1 (0.5) | 7 (2.5) | > .05 | |
| Ligament: Medial/inner collateral ligament (MCL) tear | 8 (1.6) | 4 (1.8) | 4 (1.4) | > .05 | |
| Ligament: Posterior cruciate ligament (PCL) tear | 7 (1.4) | 4 (1.8) | 3 (1.1) | > .05 | |
| Ligament: Medial/inner collateral ligament (MCL) sprain | 4 (0.8) | 2 (0.9) | 2 (0.7) | > .05 | |
| Muscle: Calf muscle injury | 4 (0.8) | 3 (1.4) | 1 (0.4) | > .05 | |
| Joint: Kneecap dislocation | 3 (0.6) | 3 (1.4) | 0 (0.0) | > .05 | |
| Ligament: Lateral/outer collateral ligament (LCL) sprain | 3 (0.6) | 2 (0.9) | 1 (0.4) | > .05 | |
| Ligament: Runner’s knee / Iliotibial ligament syndrome (inflammation on the outside of the knee due to friction between the iliotibial ligament and the outside femur) | 3 (0.6) | 2 (0.9) | 1 (0.4) | > .05 | |
| Unhappy Triad | 3 (0.6) | 0 (0.0) | 3 (1.1) | > .05 | |
| Bone: Broken tibia (lower leg) | 2 (0.4) | 0 (0.0) | 2 (0.7) | > .05 | |
| Joint: Cartilage injury | 2 (0.4) | 0 (0.0) | 2 (0.7) | > .05 | |
| Ligament: Lateral/outer collateral ligament (LCL) tear | 2 (0.4) | 0 (0.0) | 2 (0.7) | > .05 | |
| Bone edema in the femur | 1 (0.2) | 0 (0.0) | 1 (0.4) | > .05 | |
| Bone: broken patella | 1 (0.2) | 1 (0.5) | 0 (0.0) | > .05 | |
| Ligament: Posterior cruciate ligament (PCL) sprain | 1 (0.2) | 0 (0.0) | 1 (0.4) | > .05 | |
| Lower leg injury, not specified | 1 (0.2) | 0 (0.0) | 1 (0.4) | > .05 | |
| Muscle: Quadriceps tendon (thigh muscle) tear at the knee | 1 (0.2) | 1 (0.5) | 0 (0.0) | > .05 | |
| Muscle: patella tendon tear at the knee | 1 (0.2) | 0 (0.0) | 1 (0.4) | > .05 | |
| Plica Syndrome | 1 (0.2) | 0 (0.0) | 1 (0.4) | > .05 | |
| Tendon: Pes anserinus syndrome (inflammation of the tendon attachments at the lower inner side of the knee) | 1 (0.2) | 0 (0.0) | 1 (0.4) | > .05 | |
| **Ankle or Foot** | **139 (27.7)** | **58 (26.7)** | **81 (28.5)** | > .05 | |
| Ligaments: Outer/ Lateral ankle ligament tear or bony avulsion | 43 (8.6) | 16 (7.4) | 27 (9.5) | > .05 | |
| Ligaments: Ankle sprain lateral / outside | 42 (8.4) | 20 (9.2) | 22 (7.7) | > .05 | |
| Ankle injury, not specified | 16 (3.2) | 6 (2.8) | 10 (3.5) | > .05 | |
| Foot injury, not specified | 10 (2.0) | 6 (2.8) | 4 (1.4) | > .05 | |
| Bone: Broken bone of the foot | 8 (1.6) | 3 (1.4) | 5 (1.8) | > .05 | |
| Ligaments: Ankle sprain medial / inside | 4 (0.8) | 2 (0.9) | 2 (0.7) | > .05 | |
| Ligaments: Inner/ Medial ankle ligament tear or bony avulsion | 4 (0.8) | 1 (0.5) | 3 (1.1) | > .05 | |
| Bone: Broken fibula (lower leg bone at the ankle) | 3 (0.6) | 1 (0.5) | 2 (0.7) | > .05 | |
| Tendon: Achilles tendinitis (inflammation) | 3 (0.6) | 0 (0.0) | 3 (1.1) | > .05 | |
| Joint: Cartilage injury | 2 (0.4) | 1 (0.5) | 1 (0.4) | > .05 | |
| Toe injury, not specified | 2 (0.4) | 1 (0.5) | 1 (0.4) | > .05 | |
| Bone: Broken toe | 2 (0.4) | 2 (0.9) | 0 (0.0) | > .05 | |
| Tendon: Achilles tendon rupture | 1 (0.2) | 1 (0.5) | 0 (0.0) | > .05 | |
| Bone: Broken tibia (lower leg bone at the ankle) | 1 (0.2) | 0 (0.0) | 1 (0.4) | > .05 | |
| **Shoulder** | **65 (13.2)** | **22 (10.1)** | **43 (15.1)** | > .05 | |
| Tendon: SLAP-tear (tear of the upper glenoid labrum – rim around the socket – where the long head of biceps tendon attaches) | 14 (2.8) | 1 (0.5) | 13 (4.6) | > .05 | |
| Joint: Dislocated shoulder | 13 (2.6) | 6 (2.8) | 7 (2.5) | > .05 | |
| Joint: Subjectively „unstable“ shoulder / subluxation | 8 (1.6) | 4 (1.8) | 4 (1.4) | > .05 | |
| Tendon: Long head of the Biceps Tendinitis | 5 (1.0) | 2 (0.9) | 3 (1.1) | > .05 | |
| Shoulder injury, not specified | 4 (0.8) | 1 (0.5) | 3 (1.1) | > .05 | |
| Joint: Bursitis / Tendinitis of the rotator cuff | 3 (0.6) | 0 (0.0) | 3 (1.1) | > .05 | |
| Tendon: Impingement Syndrome | 3 (0.6) | 2 (0.9) | 1 (0.4) | > .05 | |
| Soft tissue: Muscle sprain/tear | 3 (0.6) | 1 (0.5) | 2 (0.7) | > .05 | |
| Muscle: Pectoralis major/minor muscle injury | 3 (0.6) | 3 (1.4) | 0 (0.0) | > .05 | |
| Joint: Sternoclavicular joint dislocation/instability (inner collar bone joint injury) | 2 (0.4) | 0 (0.0) | 2 (0.7) | > .05 | |
| Joint: Bursitis / tendinitis of the rotator cuff | 2 (0.4) | 1 (0.5) | 1 (0.4) | > .05 | |
| Bone: Broken clavicle (collarbone) | 2 (0.4) | 0 (0.0) | 2 (0.7) | > .05 | |
| Joint: Separated shoulder (acromioclavicular joint injury/dislocation) | 1 (0.2) | 1 (0.5) | 0 (0.0) | > .05 | |
| Tendon: Rotator cuff tear | 1 (0.2) | 0 (0.0) | 1 (0.4) | > .05 | |
| Soft tissue: Contusion / bruise | 1 (0.2) | 0 (0.0) | 1 (0.4) | > .05 | |
| Bone: Broken humerus (fracture of upper arm at the joint) | 1 (0.2) | 1 (0.5) | 0 (0.0) | > .05 | |
| **Hand / Wrist** | **49 (9.8)** | **29 (13.4)** | **20 (7.0)** | > .05 | |
| Bone: Broken finger | 15 (3.0) | 8 (3.7) | 7 (2.5) | > .05 | |
| Bone: Broken carpal bone at the wrist (small bones of the hand) | 7 (1.4) | 5 (2.3) | 2 (0.7) | > .05 | |
| Wrist / metacarpal injury, not specified | 6 (1.2) | 3 (1.4) | 3 (1.1) | > .05 | |
| Joint: Finger dislocation | 5 (1.0) | 4 (1.8) | 1 (0.4) | > .05 | |
| Finger injury, not specified | 4 (0.8) | 1 (0.5) | 3 (1.1) | > .05 | |
| Base of the thumb: Gamekeeper’s thumb / skier’s thumb / UCL tear (injury to the ulnar collateral ligament (UCL) | 3 (0.6) | 3 (1.4) | 0 (0.0) | > .05 | |
| Bone: Broken metacarpal bone (long bones in the palm of the hand) | 2 (0.4) | 1 (0.5) | 1 (0.4) | > .05 | |
| Bone: Broken radius (forearm bone at the wrist) | 2 (0.4) | 1 (0.5) | 1 (0.4) | > .05 | |
| Bone: Broken ulna (forearm bone at the wrist) | 1 (0.2) | 1 (0.5) | 0 (0.0) | > .05 | |
| Joint: Jammed finger | 1 (0.2) | 1 (0.5) | 0 (0.0) | > .05 | |
| Splittered saddle joint | 1 (0.2) | 0 (0.0) | 1 (0.4) | > .05 | |
| Subluxed wrist | 1 (0.2) | 0 (0.0) | 1 (0.4) | > .05 | |
| Tendon: Mallet finger / hammer finger (inability to straighten the fingertip due to an extensor tendon injury) | 1 (0.2) | 1 (0.5) | 0 (0.0) | > .05 | |
| **Hip / Pelvis / Thigh** | **28 (5.6)** | **14 (6.5)** | **14 (4.9)** | > .05 | |
| Muscle: Pulled/ Torn/ Strained hamstring muscle | 9 (1.8) | 5 (2.3) | 4 (1.4) | > .05 | |
| Hip injury, not specified | 5 (1.0) | 4 (1.8) | 1 (0.4) | > .05 | |
| Thigh injury, not specified | 5 (1.0) | 0 (0.0) | 5 (1.8) | > .05 | |
| Muscle: Avulsion / tear off from the bone of the quadriceps muscle at the hip | 2 (0.4) | 2 (0.9) | 0 (0.0) | > .05 | |
| Joint: Labral tear (cartilage rim around hip socket) | 1 (0.2) | 0 (0.0) | 1 (0.4) | > .05 | |
| Upper leg injury, not specified | 1 (0.2) | 0 (0.0) | 1 (0.4) | > .05 | |
| Pelvis injury, not specified | 1 (0.2) | 1 (0.5) | 0 (0.0) | > .05 | |
| Joint: Hip Impingement | 1 (0.2) | 0 (0.0) | 1 (0.4) | > .05 | |
| Joint: Hip dislocation | 1 (0.2) | 1 (0.5) | 0 (0.0) | > .05 | |
| Soft tissue: Piriformis syndrome (compression of the sciatic nerve by the piriformis muscle) | 1 (0.2) | 1 (0.5) | 0 (0.0) | > .05 | |
| Joint: Cartilage damage | 1 (0.2) | 0 (0.0) | 1 (0.4) | > .05 | |
| **Head and Neck** | **27 (5.4)** | **14 (6.0)** | **13 (4.6)** | > .05 | |
| Head: Broken nose | 13 (2.6) | 7 (3.2) | 6 (2.1) | > .05 | |
| Brain: Concussion | 4 (0.8) | 1 (0.5) | 3 (1.1) | > .05 | |
| Neck injury, not specified | 2 (0.4) | 1 (0.5) | 1 (0.4) | > .05 | |
| Neck: Whiplash injury | 2 (0.4) | 1 (0.5) | 1 (0.4) | > .05 | |
| Broken eyebrow | 1 (0.2) | 0 (0.0) | 1 (0.4) | > .05 | |
| Others: Chipped/broken tooth | 1 (0.2) | 0 (0.0) | 1 (0.4) | > .05 | |
| Brain: loss of consciousness (with symptoms possibly lasting for up to 2 weeks) | 1 (0.2) | 1 (0.5) | 0 (0.0) | > .05 | |
| Ear injury | 1 (0.2) | 1 (0.5) | 0 (0.0) | > .05 | |
| Neck: Disk injury | 1 (0.2) | 1 (0.5) | 0 (0.0) | > .05 | |
| **Elbow / Arm** | **17 (3.4)** | **9 (4.2)** | **8 (2.8)** | > .05 | |
| Ligament: Radial Collateral Ligament (RCL) injury | 4 (0.8) | 2 (0.9) | 2 (0.7) | > .05 | |
| Elbow injury, not specified | 4 (0.8) | 3 (1.4) | 1 (0.4) | > .05 | |
| Ligament: Thrower’s elbow (sprain injury of the ulnar collateral ligament) | 2 (0.4) | 1 (0.5) | 1 (0.4) | > .05 | |
| Tendon: Epicondylitis / tennis or golfer‘s elbow | 2 (0.4) | 1 (0.5) | 1 (0.4) | > .05 | |
| Joint: Elbow dislocation | 1 (0.2) | 1 (0.5) | 0 (0.0) | > .05 | |
| Tendon: Biceps tendonitis (at the elbow) | 1 (0.2) | 0 (0.0) | 1 (0.4) | > .05 | |
| Soft tissue: Contusion / bruise | 1 (0.2) | 0 (0.0) | 1 (0.4) | > .05 | |
| Bone: Broken radius (forearm) | 1 (0.2) | 1 (0.5) | 0 (0.0) | > .05 | |
| Bone: Broken ulna (forearm) | 1 (0.2) | 0 (0.0) | 1 (0.4) | > .05 | |
| **Spine (below Neck)** | **9 (1.8)** | **4 (1.8)** | **5 (1.8)** | > .05 | |
| Spinal nerve injury with a loss of sensation (feeling) or muscle power | 3 (0.6) | 2 (0.9) | 1 (0.4) | > .05 | |
| Muscular back pain | 2 (0.4) | 1 (0.5) | 1 (0.4) | > .05 | |
| Disk injury / prolapse | 2 (0.4) | 0 (0.0) | 2 (0.7) | > .05 | |
| Lower back injury, not specified | 2 (0.4) | 1 (0.5) | 1 (0.4) | > .05 | |
| **Chest Wall / Torso / Abdomen** | **4 (0.6)** | **3 (1.4)** | **1 (0.4)** | > .05 | |
| Bone: Rib fracture | 2 (0.4) | 1 (0.5) | 1 (0.4) | > .05 | |
| Bone: Rib contusion / bruise | 2 (0.4) | 2 (0.9) | 0 (0.0) | > .05 | |

Categorical variables are shown as number of patients and percentages per group. Bolded p-values and asterisks indicates significant difference between groups (p< .05).
