# Supplementary material for: Off‐season beach handball participation lowers injury incidence among handball players—A cross‐sectional survey on 641 athletes
Source: Knee Surg Sports Traumatol Arthrosc. 2025 Apr 18;33(6):2307–16. doi: 10.1002/ksa.12677 (PMC12104784; doi:10.1002/ksa.12677)
Supplement: Supplementary file 9 — ESM 9. [file KSA-33-2307-s002.docx]

Online Resource 9: Multivariate logistic regression for independent risk factors for injuries in indoor handball over the three-year period prior to completion of the survey

|  | | | | |
| --- | --- | --- | --- | --- |
| **Variable** | **OR** | **95% CI** | | **p-value** |
| Sex (1=male, 2=female) | 0.735 | 0.437 | 1.235 | > .05 |
| Age | 0.964 | 0.924 | 1.006 | > .05 |
| Height | 1.108 | 0.965 | 1.272 | > .05 |
| Weight | 0.895 | 0.762 | 1.051 | > .05 |
| BMI | 1.459 | 0.876 | 2.429 | > .05 |
| Years played beach handball | 1.05 | 1.007 | 1.095 | **.022*** |
| Months playing beach handball per year | 0.989 | 0.938 | 1.042 | > .05 |
| Play level (1=amateur, 2= competitive, 3= semi-professional, 4=professional) | 1.14 | 0.807 | 1.612 | > .05 |
| Competition level (1=local, 2=regional, 3=nationwide, 4 = international) | 1.101 | 0.866 | 1.399 | > .05 |
| Weeks off season | 1.012 | 0.976 | 1.048 | > .05 |
| Do you regularly play beach handball? | **0.604** | 0.421 | 0.867 | **.006*** |
| Off-season: Strength traiing | 1.399 | 0.935 | 2.093 | > .05 |
| Off-season: Flexibility training | 1.267 | 0.878 | 1.829 | > .05 |
| Off-season: Endurance training | 1.118 | 0.712 | 1.755 | > .05 |
| Off-season: Neuromuscular / proprioceptive training | 1.017 | 0.65 | 1.59 | > .05 |
| Off-season: No sports, | 1.369 | 0.681 | 2.752 | > .05 |
