# Supplementary material for: Off‐season beach handball participation lowers injury incidence among handball players—A cross‐sectional survey on 641 athletes
Source: Knee Surg Sports Traumatol Arthrosc. 2025 Apr 18;33(6):2307–16. doi: 10.1002/ksa.12677 (PMC12104784; doi:10.1002/ksa.12677)
Supplement: Supplementary file 10 — RevisedESM 10. [file KSA-33-2307-s003.docx]

Online Resource 10: Injury mechanism and distribution between beach-and-indoor handball athletes vs. indoor-only handball athletes

|  | | | | |  |
| --- | --- | --- | --- | --- | --- |
|  | **All injuries (n=501)** | Injuries of beach-and-indoor handball athletes **(n=217)** | Iniuries of indoor-only handball athletes  **(n=284)** | **p-value** | |
| **Contact**, n (%) | **195 (38.9)** | **77 (35.5)** | **118 (41.6)** | > .05 | |
| Contact: Collision with a team mate | 21 (4.2) | 7 (3.2) | 14 (4.9) | > .05 | |
| Contact: Collision with an opponent | 93 (18.6) | 35 (16.1) | 58 (20.4) | > .05 | |
| Contact: Contact with out-of-bounds apparatus | 1 (0.2) | 1 (0.5) | 0 (0.0) | > .05 | |
| Contact: Contact with the ball | 18 (3.6) | 9 (4.1) | 9 (3.2) | > .05 | |
| Contact: Contact with the floor | 18 (3.6) | 9 (4.1) | 9 (3.2) | > .05 | |
| Contact: Contact with the goal | 1 (0.2) | 0 (0.0) | 1 (0.4) | > .05 | |
| Contact: Hit by a team mate coming down after a jump | 10 (2.0) | 5 (2.3) | 5 (1.8) | > .05 | |
| Contact: Hit by an opponent coming down after a jump | 33 (6.6) | 11 (5.1) | 22 (7.7) | > .05 | |
| **Jump**, n (%) | **111 (22.2)** | **56 (25.8)** | **55 (19.4)** | > .05 | |
| Jump: Coming down on a team mate after a jump | 15 (3.0) | 9 (4.1) | 6 (2.1) | > .05 | |
| Jump: Coming down on an opponent after a jump | 29 (5.8) | 11 (5.1) | 18 (6.3) | > .05 | |
| Jump: Coming down on the floor after a jump | 67 (13.4) | 36 (16.6) | 31 (10.9) | > .05 | |
| **Playing the ball**, n (%) | **29 (5.8)** | **14 (6.5)** | **15 (5.3)** | > .05 | |
| Playing the ball: Whilst blocking | 5 (1.0) | 2 (0.9) | 3 (1.1) | > .05 | |
| Playing the ball: Whilst passing | 7 (1.4) | 3 (1.4) | 4 (1.4) | > .05 | |
| Playing the ball: Whilst throwing | 16 (3.2) | 9 (4.1) | 7 (2.5) | > .05 | |
| Playing the ball: Whilst tipping / bouncing | 1 (0.2) | 0 (0.0) | 1 (0.4) | > .05 | |
| **Other**, n (%) | **166 (33.1)** | **70 (32.3)** | **96 (33.8)** | > .05 | |
| Others: Acute overwork | 36 (7.2) | 16 (7.4) | 20 (7.0) | > .05 | |
| Others: Falling | 24 (4.8) | 14 (6.5) | 10 (3.5) | > .05 | |
| Others: Rapid change of direction | 72 (14.4) | 27 (12.4) | 45 (15.8) | > .05 | |
| Others: Rotation around planted foot | 25 (5.0) | 8 (3.7) | 17 (6.0) | > .05 | |
| Others: Unintentionally hit by ball | 2 (0.4) | 2 (0.9) | 0 (0.0) | > .05 | |
| Other, not specified | 7 (1.4) | 3 (1.4) | 4 (1.4) | > .05 | |

Categorical variables are shown as number of patients and percentages per group. Bolded p-values and asterisks indicates significant difference between groups (p< .05).
