# Supplementary material for: Off‐season beach handball participation lowers injury incidence among handball players—A cross‐sectional survey on 641 athletes
Source: Knee Surg Sports Traumatol Arthrosc. 2025 Apr 18;33(6):2307–16. doi: 10.1002/ksa.12677 (PMC12104784; doi:10.1002/ksa.12677)
Supplement: Supplementary file 11 — ESM 11. [file KSA-33-2307-s009.docx]

Online Resource 11: Injury timing and distribution between beach-and-indoor handball athletes vs. indoor-only handball athletes

|  | | | | |
| --- | --- | --- | --- | --- |
|  | All injuries (n=501) | Injuries of beach-and-indoor handball athletes (n=217) | Injuries of indoor-only handball athletes  (n=284) | p-value |
| ***Did this injury occur during training or competition?***, n (%) | | |  | > .05 |
| Competition | 268 (53.5) | 119 (54.8) | 149 (52.5) | > .05 |
| Training | 233 (46.5) | 98 (45.2) | 135 (47.5) | > .05 |
| ***When was the injury (in indoor handball)?***, n (%) | |  |  | > .05 |
| During off-season | 10 (2.0) | 5 (2.3) | 5 (1.8) | > .05 |
| During preseason | 75 (15.0) | 34 (15.7) | 41 (14.4) | > .05 |
| In the first two months of the regular season | 107 (21.4) | 43 (19.8) | 64 (22.5) | > .05 |
| In the mid break of the season | 8 (1.6) | 5 (2.3) | 3 (1.1) | > .05 |
| In the middle of the regular season | 182 (36.3) | 85 (39.2) | 97 (34.2) | > .05 |
| In the last two months of the regular season | 119 (23.8) | 45 (20.7) | 74 (26.1) | > .05 |
| ***Did this injury occur in offense or defense?*** n (%) | |  |  | > .05 |
| Defense | 131 (26.1) | 60 (27.8) | 71 (25.0) | > .05 |
| Offense | 285 (56.9) | 125 (57.6) | 160 (56.3) | > .05 |
| Does not apply | 85 (17.0) | 32 (14.8) | 53 (18.7) | > .05 |
| ***What position did you play when you got injured?*** n (%) | | |  | **.034*** |
| Back court center | 89 (17.8) | 44 (20.3) | 45 (15.8) | > .05 |
| Back court left | 107 (21.4) | 46 (21.2) | 61 (21.5) | > .05 |
| Back court right | 70 (14.0) | 33 (15.2) | 37 (13.0) | > .05 |
| Goalkeeper | 64 (12.8) | 33 (15.2) | 31 (10.9) | > .05 |
| Left wing | 55 (11.0) | 25 (11.5) | 30 (10.6) | > .05 |
| Pivot | 72 (14.4) | 18 (8.3) | 54 (19.0) | **<.001*** |
| Right wing | 44 (8.8) | 18 (8.3) | 26 (9.2) | > .05 |

Categorical variables are shown as number of patients and percentages per group. Bolded p-values and asterisks indicates significant difference between groups (p< .05).
