# Supplementary material for: Off‐season beach handball participation lowers injury incidence among handball players—A cross‐sectional survey on 641 athletes
Source: Knee Surg Sports Traumatol Arthrosc. 2025 Apr 18;33(6):2307–16. doi: 10.1002/ksa.12677 (PMC12104784; doi:10.1002/ksa.12677)
Supplement: Supplementary file 12 — ESM 12. [file KSA-33-2307-s008.docx]

Online Resource 12: Injury timing of beach-and-indoor handball athletes and distribution between beach-and-indoor handball athletes that completed a transition training

|  | | | | |
| --- | --- | --- | --- | --- |
|  | Injured beach-and-indoor handball athletes (n=217) | Injured beach-and-indoor handball athletes with transition training (n=80) | Injured beach-and-indoor handball athletes without transition training (n=137) | p-value |
| ***When was the injury (in indoor handball)?***n (%) | |  |  | > .05 |
| during off-season | 5 (2.3) | 1 (1.3) | 4 (2.9) | > .05 |
| During preseason | 34 (15.7) | 17 (21.3) | 17 (12.4) | > .05 |
| In the first two months of the regular season | 43 (19.8) | 17 (21.3) | 26 (19.0) | > .05 |
| in the mid break of the season | 5 (2.3) | 0 (0.0) | 5 (3.6) | > .05 |
| in the middle of the regular season | 85 (39.2) | 34 (42.5) | 51 (37.2) | > .05 |
| in the last two months of the regular season | 45 (20.7) | 11 (13.8) | 34 (24.8) | > .05 |

Categorical variables are shown as number of patients and percentages per group. Bolded p-values and asterisks indicates significant difference between groups (p< .05).
