# Supplementary material for: Off‐season beach handball participation lowers injury incidence among handball players—A cross‐sectional survey on 641 athletes
Source: Knee Surg Sports Traumatol Arthrosc. 2025 Apr 18;33(6):2307–16. doi: 10.1002/ksa.12677 (PMC12104784; doi:10.1002/ksa.12677)
Supplement: Supplementary file 13 — ESM 13. [file KSA-33-2307-s006.docx]

Online Resource 13: Treatment of injuries and distribution between beach-and-indoor handball athletes vs. indoor-only handball athletes

|  | | | | |
| --- | --- | --- | --- | --- |
|  | All injuries (n=501) | Injuries of beach-and-indoor handball athletes (n=217) | Injuries of indoor-only handball athletes  (n=284) | p-value |
| ***How was your injury treated? (multiple answers possible)*** n (%) | | | |  |
| Rest / Waited it out (missed training and competitions) | 319 (63.7) | 147 (67.7) | 172 (60.6) | > .05 |
| Regular painkillers | 160 (31.9) | 55 (25.3) | 105 (37.0) | **<.001*** |
| Anti-inflammatory medication | 66 (13.2) | 31 (14.3) | 35 (12.3) | > .05 |
| Injections/ infiltrations (e.g. into torn muscle or into inflamed area) | 28 (5.6) | 14 (6.5) | 14 (4.9) | > .05 |
| Physiotherapy | 299 (59.7) | 134 (61.8) | 165 (58.1) | > .05 |
| Stabilization in a brace | 126 (25.1) | 45 (20.7) | 81 (28.5) | > .05 |
| Immobilization in a splint | 45 (9.0) | 20 (9.2) | 25 (8.8) | > .05 |
| Immobilization in a cast | 35 (7.0) | 35 (5.1) | 24 (8.5) | > .05 |
| Surgery | 161 (32.1) | 56 (25.8) | 105 (37.0) | **.006*** |
| **Knee / Calf / Lower leg**, n (%) |  |  |  | > .05 |
| Ligament: Anterior cruciate ligament (ACL) sprain | 7 (1.4) | 1 (0.5) | 6 (2.1) | > .05 |
| Tendon: Patellar tendinitis/ jumper’s knee | 5 (1.0) | 1 (0.5) | 4 (1.4) | > .05 |
| Ligament: Anterior cruciate ligament (ACL) tear | 48 (9.6) | 18 (8.3) | 30 (10.6) | > .05 |
| Knee injury, not specified | 4 (0.8) | 1 (0.5) | 3 (1.1) | > .05 |
| Joint: Torn meniscus | 20 (4.0) | 8 (3.7) | 12 (4.2) | > .05 |
| Ligament: Medial/inner collateral ligament (MCL) tear | 2 (0.4) | 0 (0.0) | 2 (0.7) | > .05 |
| Unhappy triad | 2 (0.4) | 0 (0.0) | 2 (0.7) | > .05 |
| Ligament: Lateral/outer collateral ligament (LCL) sprain | 2 (0.4) | 1 (0.5) | 1 (0.4) | > .05 |
| Ligament: Posterior cruciate ligament (PCL) tear | 2 (0.4) | 1 (0.5) | 1 (0.4) | > .05 |
| Joint: Kneecap dislocation | 2 (0.4) | 2 (0.9) | 0 (0.0) | > .05 |
| Ligament: Medial/inner collateral ligament (MCL) sprain | 1 (0.2) | 0 (0.0) | 1 (0.4) | > .05 |
| Patellaruptur | 1 (0.2) | 0 (0.0) | 1 (0.4) | > .05 |
| Joint: Cartilage injury Knee | 1 (0.2) | 0 (0.0) | 1 (0.4) | > .05 |
| **Ankle or Foot**, n (%) |  |  |  | > .05 |
| Ligaments: Ankle sprain lateral / outside | 3 (0.6) | 0 (0.0) | 3 (1.1) | > .05 |
| Ligament: Lateral/outer collateral ligament (LCL) tear | 2 (0.4) | 0 (0.0) | 2 (0.7) | > .05 |
| Ankle injury, not specified | 2 (0.4) | 0 (0.0) | 2 (0.7) | > .05 |
| Ligaments: Ankle sprain medial / inside | 1 (0.2) | 0 (0.0) | 1 (0.4) | > .05 |
| Ligaments: Outer/ Lateral ankle ligament tear or bony avulsion | 1 (0.2) | 0 (0.0) | 1 (0.4) | > .05 |
| Achilles | 1 (0.2) | 1 (0.5) | 0 (0.0) | > .05 |
| Bone: Broken fibula (lower leg bone at the ankle) | 1 (0.2) | 0 (0.0) | 1 (0.4) | > .05 |
| Stress fractor in the big toe | 1 (0.2) | 1 (0.5) | 0 (0.0) | > .05 |
| Joint: Cartilage injury Ankle | 1 (0.2) | 1 (0.5) | 0 (0.0) | > .05 |
| Foot injury, not specified | 1 (0.2) | 1 (0.5) | 0 (0.0) | > .05 |
| **Shoulder**, n (%) |  |  |  | > .05 |
| Tendon: SLAP-tear (tear of the upper glenoid labrum - rim around the socket - where the long head of biceps tendon attaches) | 9 (1.8) | 0 (0.0) | 9 (3.2) | > .05 |
| Joint: Dislocated shoulder | 6 (1.2) | 2 (0.9) | 4 (1.4) | > .05 |
| Bone: Broken clavicle (collarbone) | 2 (0.4) | 0 (0.0) | 2 (0.7) | > .05 |
| Tendon: Long head of the Biceps Tendinitis | 2 (0.4) | 1 (0.5) | 1 (0.4) | > .05 |
| Joint: Subjectively „unstable“ shoulder / subluxation (Glenoid Labrum Tear) | 2 (0.4) | 1 (0.5) | 1 (0.4) | > .05 |
| Tendon: Rotator cuff tear | 1 (0.2) | 0 (0.0) | 1 (0.4) | > .05 |
| Joint: Sternoclavicular joint dislocation/instability (inner collar bone joint injury) | 1 (0.2) | 0 (0.0) | 1 (0.4) | > .05 |
| Tendon: Impingement Syndrome | 1 (0.2) | 1 (0.5) | 0 (0.0) | > .05 |
| Bone: Broken humerus (fracture of upper arm at the joint) | 1 (0.2) | 1 (0.5) | 0 (0.0) | > .05 |
| **Hand / Wrist** |  |  |  | > .05 |
| Bone: Broken finger | 4 (0.8) | 3 (1.4) | 1 (0.4) | > .05 |
| Bone: Broken carpal bone at the wrist (small bones of the hand) | 2 (0.4) | 1 (0.5) | 1 (0.4) | > .05 |
| Splittered saddle joint | 1 (0.2) | 0 (0.0) | 1 (0.4) | > .05 |
| Finger injury, not specified | 1 (0.2) | 0 (0.0) | 1 (0.4) | > .05 |
| Tendon: Mallet finger / hammer finger (inability to straighten the fingertip due to an extensor tendon injury) | 1 (0.2) | 1 (0.5) | 0 (0.0) | > .05 |
| Bone: Broken radius (forearm bone at the wrist) | 1 (0.2) | 1 (0.5) | 0 (0.0) | > .05 |
| **Hip / Pelvis / Thigh** |  |  |  | > .05 |
| Hip Impingement | 1 (0.2) | 0 (0.0) | 1 (0.4) | > .05 |
| Joint: Labral tear (cartilage rim around hip socket) | 1 (0.2) | 0 (0.0) | 1 (0.4) | > .05 |
| Joint: Cartilage damage Hip | 1 (0.2) | 0 (0.0) | 1 (0.4) | > .05 |
| **Head and Neck** |  |  |  | > .05 |
| Head: Broken nose | 6 (1.2) | 2 (0.9) | 4 (1.4) | > .05 |
| Broken eyebrow | 1 (0.2) | 0 (0.0) | 1 (0.4) | > .05 |
| Ear injury | 1 (0.2) | 1 (0.5) | 0 (0.0) | > .05 |
| **Elbow / Arm** |  |  |  | > .05 |
| Elbow injury, not specified | 2 (0.4) | 2 (0.9) | 0 (0.0) | > .05 |
| Ligament: Radial Collateral Ligament (RCL) injury | 1 (0.2) | 1 (0.5) | 0 (0.0) | > .05 |
| Bone: Broken radius (forearm) | 1 (0.2) | 1 (0.5) | 0 (0.0) | > .05 |
| **Spine (below Neck)** |  |  |  | > .05 |
| Spinal nerve injury with a loss of sensation (feeling) or muscle power | 2 (0.4) | 1 (0.5) | 1 (0.4) | > .05 |
| **Chest Wall / Torso / Abdomen** | 0 | 0 | 0 | > .05 |

Categorical variables are shown as number of patients and percentages per group. Bolded p-values and asterisks indicates significant difference between groups (p< .05).
