# Supplementary material for: Off‐season beach handball participation lowers injury incidence among handball players—A cross‐sectional survey on 641 athletes
Source: Knee Surg Sports Traumatol Arthrosc. 2025 Apr 18;33(6):2307–16. doi: 10.1002/ksa.12677 (PMC12104784; doi:10.1002/ksa.12677)
Supplement: Supplementary file 14 — ESM 14. [file KSA-33-2307-s004.docx]

Online Resource 14: Return to sports and distribution between beach-and-indoor handball athletes vs. indoor-only handball athletes

|  | | | | | |  |  |
| --- | --- | --- | --- | --- | --- | --- | --- |
|  | All injuries (n=501) | Injuries of beach-and-indoor handball athletes (n=217) | Injuries of indoor-only handball athletes  (n=284) | | p-value |  |  |
| ***At the time when this injury was worst, did you have any difficulties in normal training and competition due to the injury?*** n (%) | | |  | > .05 | | |  |
| Full participation without health problems | 34 (6.8) | 20 (9.2) | 14 (4.9) | | > .05 |  |  |
| Full participation, but with symptoms from the injury | 63 (12.6) | 24 (11.1) | 39 (13.7) | | > .05 |  |  |
| Reduced participation due to the injury | 83 (16.6) | 36 (16.6) | 47 (16.5) | | > .05 |  |  |
| Could not participate due to the injury | 321 (64.1) | 137 (63.1) | 184 (64.8) | | > .05 |  |  |
| ***Have you returned to playing indoor handball since your injury?*** n (%) | |  |  | > .05. | | | |
| Yes | 436 (87.0) | 189 (87.1) | 247 (87.0) | | > .05 |  |  |
| No | 65 (13.0) | 28 (12.9) | 37 (13.0) | | > .05 |  |  |
| ***What has kept you from going back?*** n (%) | | |  | | > .05 |  |  |
| Recent injuriy | 48 (9.6) | 24 (11.1) | 24 (8.5) | | > .05 |  |  |
| Unable to regain previous form | 2 (0.4) | 0 (0.0) | 2 (0.7) | | > .05 |  |  |
| Fear of getting injured again | 3 (0.6) | 0 (0.0) | 3 (1.1) | | > .05 |  |  |
| Pain | 8 (1.6) | 3 (1.4) | 5 (1.8) | | > .05 |  |  |
| Persisting instability | 2 (0.4) | 0 (0.0) | 2 (0.7) | | > .05 |  |  |
| None of the above | 2 (0.4) | 1 (0.5) | 1 (0.4) | | > .05 |  |  |
| ***How long did it take to return to full handball training/ competition?*** n (%) | | 189 | 247 | > .05 | | | |
| 1 – 4 weeks | 124 (28.4) | 70 (37.0) | 54 (21.9) | | **<.001*** |  |  |
| ~ 2 months | 100 (22.9) | 40 (21.2) | 60 (24.3) | | > .05 |  |  |
| ~ 3 months | 55 (12.6) | 27 (14.3) | 28 (11.3) | | > .05 |  |  |
| ~ 4 months | 24 (5.5) | 8 (4.2) | 16 (6.5) | | > .05 |  |  |
| ~ 5 months | 11 (2.5) | 3 (1.6) | 8 (3.2) | | > .05 |  |  |
| ~ 6 months | 20 (4.6) | 7 (3.7) | 13 (5.3) | | > .05 |  |  |
| ~ 7 months | 10 (2.3) | 1 (0.5) | 9 (3.6) | | > .05 |  |  |
| ~ 8 months | 7 (1.6) | 3 (1.6) | 4 (1.6) | | > .05 |  |  |
| ~ 9 months | 14 (3.2) | 6 (3.2) | 8 (3.2) | | > .05 |  |  |
| ~ 10 months | 9 (2.1) | 3 (1.6) | 6 (2.4) | | > .05 |  |  |
| ~ 11 months | 3 (0.7) | 1 (0.5) | 2 (0.8) | | > .05 |  |  |
| ~ 12 months | 8 (1.8) | 3 (1.6) | 5 (2.0) | | > .05 |  |  |
| > 1 year | 23 (5.3) | 7 (3.7) | 16 (6.5) | | > .05 |  |  |
| “I did not return yet, but I think I will” | 2 (0.5) | 1 (0.5) | 1 (0.4) | | > .05 |  |  |
| “I did not return and I don’t think I will” | 2 (0.5) | 0 (0.0) | 2 (0.8) | | > .05 |  |  |

Categorical variables are shown as number of patients and percentages per group. Bolded p-values and asterisks indicates significant difference between groups (p< .05).
